# Cardiovascular complications in acute dengue infection: a population-based cohort study

**DOI:** 10.1016/j.lanwpc.2025.101713

**Published:** 2025-10-16

**Authors:** Liang En Wee, Wei Zhi Tan, Jo Yi Chow, Jue Tao Lim, Calvin Chiew, Po Ying Chia, Lee Ching Ng, Mohammed Rizwan Amanullah, Jonathan Yap, Khung Kheong Yeo, Mark Yan Yee Chan, Derek J. Hausenloy, Yee Sin Leo, David Chien Lye, Kelvin Bryan Tan

**Affiliations:** a Communicable Diseases Agency, Singapore; b Duke-NUS Medical School, National University of Singapore, Singapore; c Department of Infectious Diseases, Singapore General Hospital, Singapore; d Lee Kong Chian School of Medicine, Nanyang Technological University, Singapore; e National Centre for Infectious Diseases, Singapore; f Department of Infectious Diseases, Tan Tock Seng Hospital, Singapore; g Environmental Health Institute, National Environment Agency, Singapore; h Saw Swee Hock School of Public Health, National University of Singapore, Singapore; i School of Biological Sciences, Nanyang Technological University, Singapore; j Department of Cardiology, National Heart Centre Singapore, Singapore; k Yong Loo Lin School of Medicine, National University of Singapore, Singapore; l Department of Cardiology, National University Heart Centre, Singapore; m National Heart Research Institute Singapore, National Heart Centre Singapore, Singapore; n The Hatter Cardiovascular Institute, University College London, London, United Kingdom; o Ministry of Health, Government of Singapore, Singapore

**Keywords:** Dengue, Cardiovascular, Acute, Atrial fibrillation, Dysrhythmia

## Abstract

**Background:**

Acute dengue-virus (DENV) infection, while associated with mild disease in most instances, is anecdotally associated with more severe cardiovascular complications. However, more accurate estimates of cardiac complications are required to evaluate the actual burden of dengue-associated morbidity, given significant contribution of cardiovascular events to overall morbidity and rising incidence of DENV-infection attributable to climate change.

**Methods:**

A population-based cohort of DENV-infected Singaporean adults (2017–2023) and population-based controls (without DENV-infection) was constructed using the national-dengue-registry. Overlap weights were employed to balance baseline covariates. Logistic regression was used to estimate odds of cardiovascular events in DENV-infected cases versus uninfected controls up to 30 days from T_0_ (notification-date in DENV-infected adults; randomly assigned in population-based controls following distribution of T_0_ amongst DENV-infected cases).

**Findings:**

65,207 DENV-infected cases and 1,616,865 uninfected controls were included. Higher odds of any cardiovascular event (adjusted-odds-ratio, aOR = 10.63 [95% CI = 7.56, 15.48]), major-adverse-cardiac-event (MACE) (aOR = 2.92 [95% CI = 1.81–4.88]), dysrhythmia (aOR = 18.44 [95% CI = 11.25 = 32.96]) and ischemic-heart-disease (aOR = 3.00 [95% CI = 1.83–5.15]) were observed up to 30 days post-DENV-infection, versus uninfected controls. Odds of acute cardiovascular events remained higher in both ambulatory/hospitalised DENV-infected cases, DENV-IgG-positive/negative cases, and across DENV1/2 and DENV3-predominant transmission, versus uninfected controls. However, overall excess burden (EB) of acute cardiovascular events in DENV-infected adults was modest, with <1 excess event per-100-cases except amongst those aged ≥60 years (EB = 1.25 [95% CI = 1.05–1.44]).

**Interpretation:**

Acute DENV-infection was associated with higher odds of cardiovascular events up to 30 days post-infection; though excess-burden was modest. Older adults at higher risk should be monitored for cardiac complications following acute DENV-infection.

**Funding:**

National-Medical-Research-Council, Singapore.

**Research in context**

**Evidence before this study**

We conducted a Pubmed search up to 1st August 2025 using the following search terms: “dengue”, “cardiac”, “cardiovascular”, “population”. Findings of association between cardiovascular events and dengue virus (DENV)-infection were limited for the most part to case-reports and small prospective cohorts of hospitalised DENV patients, given dengue's status as a neglected tropical disease and limited follow-up for mildly-infected individuals in under-resourced settings. Only two population-based retrospective cohort studies found increased risk of major-adverse-cardiovascular-events (MACE) and heart failure, respectively, up to 30 days following DENV-infection; but generalisability was limited as both studies were conducted in the same population using a common data source (administrative claims), and a self-controlled-case-series design restricted analysis to DENV-infected individuals only, limiting population-wide estimates of the excess burden of cardiovascular events attributable to DENV-infection.

**Added value of this study**

Odds of a comprehensive spectrum of cardiovascular events (including ischemic-heart-disease, dysrhythmias, inflammatory-heart-disease, and thrombotic conditions) following acute DENV-infection were estimated using a retrospective population-based cohort study design in the context of a tropical Southeast Asian city-state where DENV-infection is endemic and diagnostic testing widely available across healthcare settings. Elevated odds of any cardiovascular event, major-adverse-cardiac-event (MACE), dysrhythmia and ischemic-heart-disease were observed up to 30 days post-DENV-infection, versus uninfected controls. Odds of acute cardiovascular events remained elevated in both ambulatory/hospitalised DENV-infected cases, DENV-IgG-positive/negative cases, and across DENV1/2 and DENV3-predominant transmission, versus uninfected controls. However, overall excess burden (EB) of acute cardiovascular events in DENV-infected adults was modest, with <1 excess event per-100-cases, except amongst those aged ≥60 years.

**Implications of all the available evidence**

DENV-infection was associated with significantly higher odds of cardiovascular events following acute infection; though excess-burden was modest. Older adults at higher risk should be monitored for cardiac complications following acute DENV-infection.

## Introduction

Dengue virus (DENV)-infection is a vector-borne viral infection that accounts for a significant global burden of disease, particularly in low-and-middle-income-countries (LMICs) and in endemic tropical regions.^1^ While DENV-infection is usually associated with mild and self-limiting infection, severe manifestations, such as acute cardiovascular events, can occur^2^; however, estimates of prevalence vary widely. In a systematic review of acute cardiovascular sequelae following DENV-infection that pooled data from ∼7000 patients, slightly under half exhibited at least one cardiac manifestation^3^; whereas in small cohorts of hospitalised DENV patients evaluated prospectively via serial biomarkers and imaging for cardiac complications, only 15–25% exhibited evidence of cardiac involvement.^4^, ^5^, ^6^ More accurate estimates of cardiac complications are required to evaluate the actual burden of DENV-associated morbidity and justify the benefit of public-health strategies including therapeutics and vaccine development, given significant contribution of cardiovascular events to overall morbidity and resurgence of DENV-infection due to global warming and climate change.^7^

In addition, most existing epidemiological studies on the burden of DENV-infection and cardiovascular complications are significantly limited by the inclusion of hospitalised DENV-infected cases only.^4^, ^5^, ^6^ The majority of DENV-infected individuals do not require hospitalisation; in under-resourced settings, testing and follow-up data for mildly-infected individuals is usually unavailable given restricted access to diagnostic testing for DENV-infection, limiting population-wide estimates of acute cardiovascular sequelae. Two population-based cohort studies in Taiwanese adults utilising the same national healthcare-claims database found increased risk of major-adverse-cardiovascular-events (MACE) and heart failure up to 30 days following DENV-infection, with highest risk in the first two weeks post-infection.^8^^,^^9^ However, use of a self-controlled-case-series design for evaluation of cardiovascular events limited analysis to DENV-infected individuals only.^8^^,^^9^ We utilised national registries of DENV-infection, fused with national healthcare-claims data, to evaluate risks and excess burden (EB) of acute cardiovascular events in a national population-based cohort of DENV-infected adults up to 30 days post-infection, versus uninfected controls. To provide additional context on the potential impact of DENV-infection on acute cardiac risk, versus other viral infections, risk of acute cardiovascular events following DENV-infection was additionally contrasted against that following COVID-19, given co-circulation of DENV-infection with COVID-19 in tropical/subtropical regions during endemicity.^10^

## Methods

### Study setting and databases

Singapore is a multi-ethnic tropical Southeast Asian city-state (population: 6.03 million), where DENV-infection is endemic. DENV-infection is legally notifiable to the local Ministry-of-Health (MOH) not later than 24 h from clinical diagnosis.^10^ Confirmatory diagnostic testing for DENV-infection is widely available across healthcare settings in Singapore, with rapid-diagnostic-tests widely utilised in primary-care and additional tests (eg. polymerase-chain-reaction [PCR]/enzyme-linked-immunoassay [ELISA]) available in hospitals.^10^ Testing results are requested at point-of-notification.^10^ As such, national registries were utilised to construct a cohort of all adult Singaporeans with laboratory-confirmed DENV-infection, as well as a cohort of population-based controls without evidence of DENV-infection.

Risk of acute cardiovascular events was assessed using the national healthcare-claims database (Mediclaims). In Singapore, the national government-administered medical-savings scheme (Medisave) and healthcare-insurance scheme (Medishield) can be claimed against for inpatient care and outpatient treatment at both public and private healthcare providers; participation is compulsory.^11^ This enabled comprehensive capture of cardiovascular events across different healthcare settings. Previously, the Mediclaims database has been utilised to evaluate acute cardiovascular complications following other viral infections (eg. SARS-CoV-2), in our local population^12^; as well as risks of cardiovascular sequelae in the post-acute setting following DENV-infection.^13^^,^^14^

### Cohort

**Fig. 1:**
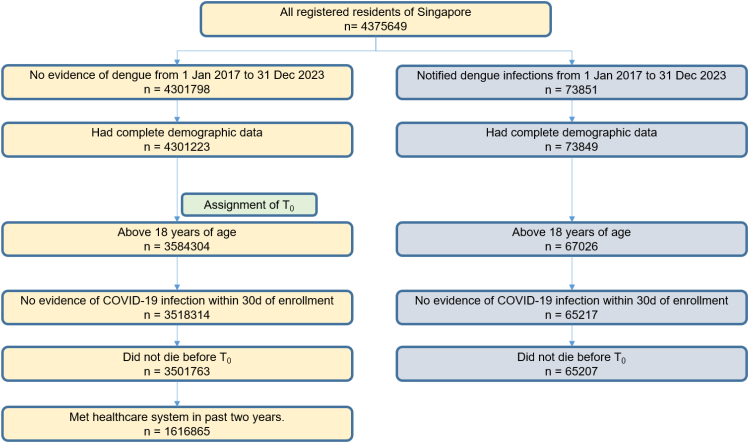
Cohort construction flowchart, for DENV-infected cases and DENV-uninfected population-based controls. T_0_, index-date. For the DENV-infected cohort, T_0_ was taken as date-of-test; for DENV-uninfected population-based controls, T_0_ was randomly assigned according to distribution of T_0_ amongst test-positives.

A retrospective population-based cohort study design was utilised; a flowchart of cohort construction for DENV-infected individuals and population-based controls without evidence of DENV-infection is provided in Fig. 1. Adult Singaporean citizens/permanent residents aged ≥18 years, without missing sociodemographic information, and who were alive as of the index-date, T_0_, were enrolled from 1st January 2017 to 31st December 2023. For the DENV-infected cohort, T_0_ was taken as date-of-test; for population controls with no evidence of DENV-infection from 1st January 2017 to 31st December 2023, T_0_ was randomly assigned according to distribution of T_0_ amongst test-positives,^14^ to generate a comparator group that had a similar distribution of length-of-follow-up. In these cohorts, given subsequent emergence of COVID-19 in the later part of the study period, and known elevated risk of acute cardiovascular complications following SARS-CoV-2 infection,^12^ individuals with documented SARS-CoV-2 infection within 30 days of T_0_ were additionally excluded. SARS-CoV-2 infection status was classified using the national COVID-19 registry.^10^^,^^12^, ^13^, ^14^ For uninfected population-based controls, to ensure that these individuals were still in contact with the healthcare system and available for follow-up, individuals without any Mediclaims utilization (outpatient/inpatient visit) in the past 2 years prior to T_0_ were additionally excluded.

### Pre-specified cardiovascular outcomes

Acute new-incident cardiovascular events following DENV-infection were assessed over the acute (T_0 -_T_0_+30) follow-up period. A 30-day follow-up period was utilized in light of prior population-based cohort studies that found elevated risk of major-adverse-cardiovascular/cerebrovascular-event (MACE) and heart failure within the first two weeks of DENV-infection when utilising a self-controlled case-series design.^8^^,^^9^ No significant risk of new-onset MACE was observed after two weeks post-DENV-infection,^8^ and the incidence-rate-ratio of new-onset heart failure was substantially diminished in the third and fourth weeks following DENV-infection.^9^ Additionally, in a hospitalised cohort of DENV-infected adults prospectively evaluated for cardiac involvement with serial echocardiography, improving trends in parameters such as left-ventricular-ejection-fraction (LVEF), stroke-volume and cardiac index were already observed during early convalescence (<2 weeks post-discharge).^6^ Cardiovascular diagnoses were based on International-Classification-of-Diseases, Tenth-Revision (ICD-10) codes recorded in Mediclaims, referencing previously published work on acute/post-acute cardiovascular sequelae following DENV-infection in population-based studies.^8^^,^^9^^,^^13^^,^^14^ In order to identify new-incident cardiovascular events, for each specific cardiovascular event, individuals with a history of the specific event recorded in Mediclaims within the past 5 years were excluded. Any acute cardiovascular event was taken as a composite of any ischemic-heart-disease, dysrhythmias, inflammatory-heart-disease, other cardiac disease (eg. cardiomyopathy/cardiogenic shock), and thrombotic conditions.^8^^,^^9^^,^^13^^,^^14^ Major-adverse-cardiovascular/cerebrovascular-event (MACE) was defined as the first incidence of myocardial infarction, ventricular arrhythmia, or sudden-cardiac-death.^8^ Specific cardiovascular events with higher incidence post-DENV-infection reported in prior literature were individually evaluated: these included ischemic-heart-disease,^8^ and dysrhythmia.^13^^,^^14^ Other categories of cardiovascular events were not individually evaluated but included in the composite of acute events, due to paucity of new-incident cases. Relevant ICD-10 codes are detailed in the Supplementary Material (pg. 16–19).

### Covariates

Demographics (age, sex, ethnicity), comorbidities (Charlson-Comorbidity-Index [CCMI] and cardiovascular risk factors including hypertension/diabetes/dyslipidemia), prior healthcare utilisation, and socioeconomic-status (SES), defined at the point of T_0_ were included as covariates; these factors were included as they were known risk factors for cardiovascular events in our population,^15^ and corresponding information was available in national electronic-health-records. As missing demographic data was minimal due to availability and usage of national electronic-health-records/databases maintained by the MOH, individuals with missing demographic data were dropped from analysis; missing data was not imputed.

### Statistical analysis

Risks of pre-specified new-incident acute cardiovascular events post-DENV-infection (0–30 days from T_0_) were estimated using a comparator group of individuals with no evidence of DENV-infection. Baseline characteristics of comparator and DENV-infected groups, along with standardised-mean-differences (SMD), were computed, with proportions presented for categorical variables and means/standard-deviations presented for continuous variables. Overlap weighting was utilised to adjust for differences in baseline characteristics and ensured covariate balance between DENV-infected and DENV-uninfected groups post-weighting.^16^ All observable characteristics available in the dataset were adjusted for. By down-weighting units in the tails of the propensity score distribution, overlap weights address the potential issue of extreme propensity scores resulting in biased estimates.^16^ Overlap weights were taken as equal to the propensity score if DENV-uninfected and 1-propensity score if DENV-infected. Propensity scores were estimated taking DENV-infection as the outcome variable and all available covariates as explanatory variables. SMD <0.1 was taken as the threshold for good covariate balance post-weighting.

For estimation of risks in the acute phase, odds-ratios (ORs) of cardiovascular events between the DENV-infected and comparator groups were estimated using logistic regression, with overlap weights applied. ORs were taken as an approximation of risks, given that cardiovascular outcomes were rare. Acute excess burdens (EBs) per-100 persons were defined and computed as the difference in weighted incidence rates of cardiac complications in DENV-infected and comparator groups. DENV-infected cases were further stratified into those with hospitalisation attributed to DENV (defined as hospitalisation with a corresponding ICD-10 code for DENV-infection [A90/A91] recorded in Mediclaims within 30 days of test-positive date), and those without hospitalisation attributed to DENV; possible secondary DENV-infection cases (defined as positive DENV-IgG at the point-of-notification, in the subset of DENV-infected cases where DENV-IgG was tested for) versus DENV-IgG-negative cases; and cases infected during DENV1/2 and DENV3-predominant transmission, with information on predominant circulating serotype derived from national genomic surveillance.^10^ To assess effect modification, interaction terms for age, gender, ethnicity, and comorbidities were initially included in exploratory analyses; as all interaction terms were not statistically significant, suggesting an absence of effect modification, with the exception of age (p < 0.001), interaction terms were removed from the final models and only subgroup analyses by age group (40–59/≥60 years) were presented.

As part of sensitivity analyses, (**1**) the use of inverse-probability-weights, together with covariate adjustment in the logistic regression step (doubly-robust) were explored as alternative weighting schemes in the main analysis. In the doubly-robust method, incorporating covariates used for the propensity scoring model in the outcome regression potentially reduces the possibility of misspecification in either the outcome or propensity scoring model; while inverse-probability-weighting assigns more weight to individuals under-represented in each group, at the potential expense of biasing estimates due to extreme propensity scores. We therefore sought to evaluate if estimates of acute cardiovascular risk post-DENV-infection remained robust when different weighting schemes were utilised. **(2)** Given high diagnostic accuracy (sensitivity/specificity) of NS-1 testing for acute dengue diagnosis,^17^ only DENV-infected cases recorded in the national dengue registry with corresponding NS-1 positivity were contrasted against DENV-uninfected controls, to assess if estimates remained robust. **(3)** Risk of new-onset acute-myocardial-infarction (AMI) in DENV-infected cases versus DENV-uninfected controls, originally defined using corresponding ICD-10 codes in national healthcare-claims data (Mediclaims), was alternatively assessed using records from the Singapore-Myocardial-Infarction-Registry (SMIR), a national registry of all patients diagnosed with AMI in Singapore, in which AMI cases were verified by trained personnel from discharge records, reimbursement claims, death registry information, and screening of cases with troponin levels elevated above the 99th percentile.^15^ For this sensitivity analysis, the study period was limited to 1st Jan 2017–31st Dec 2021, corresponding to the period of SMIR data availability. **(4)** Though follow-up time was relatively short (30-days), Cox regression, with overlap weights applied, was utilised to evaluate the hazard-ratio of new-incident cardiovascular events; to evaluate if estimates changed compared to logistic regression. **(5)** To contextualise risks of acute cardiovascular complications post-DENV-infection, versus other viral infections, odds of acute cardiovascular events post-DENV-infection were further contrasted against SARS-CoV-2-infected individuals, using similar methodology as the main analysis. COVID-19 was chosen as a comparator because **a)** acute cardiovascular complications post-COVID-19 are well-documented;^14^ **b)** during the COVID-19 pandemic, SARS-CoV-2 diagnostic testing was also widely available across healthcare settings, and COVID-19, like dengue, was also legally notifiable within 24-h of clinical diagnosis.^18^ A flowchart of cohort construction (COVID-19 cases) is provided in Supplementary Figure S1; inclusion/exclusion criteria mirrored that for the dengue-infected cohort, and the same analytical strategy of weighted regression models and excess burden calculation as above was employed. (**5)** Risks of acute bronchitis were evaluated as a negative-outcome-control; given that DENV-infection does not involve the respiratory system, risks of acute respiratory sequelae were not expected to significantly differ in DENV-infected cases versus uninfected controls. **(6)** E-values were calculated to ascertain the level of unmeasured confounding for unknown confounders required to render the risk of cardiovascular outcomes in the acute phase of dengue infection insignificant.^19^ 95% confidence-intervals (CI) were reported; a 95% CI that excluded one indicated statistical-significance. Analyses were conducted using R (version 4.3.1).

### Ethics approval

This study was undertaken as national public-health-research under the Infectious-Diseases-Act, Singapore; as such, separate ethics review by an Institutional Review Board was not required under the Act, and requirement for informed consent was waived as all study data was anonymised.

### Role of the funding source

All funders had no role in study design, data collection, data analysis, interpretation, or writing of the report.

## Results

In total, 65,207 Singaporean adults with documented laboratory-confirmed DENV-infection from 2017 to 2023 were compared against 1,616,865 contemporaneous population-based controls with no evidence of DENV-infection (Fig. 1); a very small minority were excluded because of missing demographic data (DENV-infected: N = 2; DENV-uninfected: N = 575). Demographic and clinical characteristics of both groups are presented in Table 1; after weighting, differences in baseline characteristics were minimal with SMDs<0.05. Less than a third (28.8%, 18,786/65,207) of DENV-infected cases were hospitalised.

**Table 1:** Baseline characteristics of dengue-infected Singaporean adults and population-based controls without dengue, with standardised-mean-differences before and after overlap weighting.

Variables	Controls without dengue (N = 1,616,865)	Dengue cases (N = 65,207)	Standardised-mean-difference, baseline	Controls without dengue, weighted	Dengue cases, weighted	Standardised-mean-difference, post-weighting^b^
**Age, years (S.D)**	54.84 (18.32)	48.43 (17.83)	0.36	48.8 (18.16)	48.8 (17.85)	0.00
**Age distribution, years**						
18–39 years	421,808 (26.09%)	23,858 (36.59%)	0.22	23,182 (37.8%)	21,963 (35.81%)	0.00
40–59 years	497,999 (30.80%)	23,584 (36.17%)	0.11	19,797 (32.28%)	22,225 (36.24%)	0.00
≥60 years	697,058 (43.11%)	17,765 (27.24%)	0.35	18,354 (29.93%)	17,145 (27.95%)	0.00
**Gender**						
Male	730,702 (45.19%)	34,876 (53.49%)	0.17	32,489 (52.97%)	32,489 (52.97%)	0.00
Female	886,163 (54.81%)	30,331 (46.51%)	0.17	28,845 (47.03%)	28,845 (47.03%)	0.00
**Ethnicity**						
Chinese	1,231,706 (76.18%)	51,838 (79.50%)	0.08	48,605 (79.25%)	48,605 (79.25%)	0.00
Malay	135,391 (8.37%)	5299 (8.13%)	0.01	5017 (8.18%)	5017 (8.18%)	0.00
Indian	209,114 (12.93%)	6192 (9.50%)	0.12	5960 (9.72%)	5960 (9.72%)	0.00
Others^a^	40,654 (2.51%)	1878 (2.88%)	0.02	1752 (2.86%)	1752 (2.86%)	0.00
**Housing type**						
1-2 room public housing	92,039 (5.69%)	2495 (3.83%)	0.10	2415 (3.94%)	2415 (3.94%)	0.00
3-room public housing	264,208 (16.34%)	8438 (12.94%)	0.10	8098 (13.2%)	8098 (13.2%)	0.00
4-room public housing	536,796 (33.20%)	16,720 (25.64%)	0.17	16,072 (26.2%)	16,072 (26.2%)	0.00
5-room public housing	611,955 (37.85%)	25,419 (38.98%)	0.02	24,125 (39.33%)	24,125 (39.33%)	0.00
Private housing/Others	111,867 (6.92%)	12,135 (18.61%)	0.31	10,623 (17.32%)	10,623 (17.32%)	0.00
**Comorbidity burden (Charlson comorbidity index, CCMI)**^c^						
No comorbidities (CCMI = 0)	1,149,710 (71.11%)	54,936 (84.25%)	0.36	51,312 (83.66%)	51,312 (83.66%)	0.00
Mild comorbidity burden (CCMI 1–3)	402,062 (24.87%)	8442 (12.95%)	0.35	8793 (14.34%)	8231 (13.42%)	0.00
Moderate-severe comorbidity burden (CCMI >3)	65,093 (4.03%)	1829 (2.80%)	0.07	1229 (2%)	1791 (2.92%)	0.00
**Comorbidities**						
Diabetes	19,258 (1.19%)	360 (0.55%)	0.08	353 (0.57%)	353 (0.57%)	0.00
Dyslipidemia	410,751 (25.40%)	8199 (12.57%)	0.38	8021 (13.08%)	8021 (13.08%)	0.00
Ischemic heart disease	4928 (0.30%)	170 (0.26%)	0.01	164 (0.27%)	164 (0.27%)	0.00
Cerebrovascular disease	56,139 (3.47%)	1396 (2.14%)	0.09	1357 (2.21%)	1357 (2.21%)	0.00
**Healthcare utilisation**						
Prior hospitalisation/emergency department visit in the past 1 year	1,086,302 (67.19%)	36,462 (55.92%)	0.23	34,929 (56.95%)	34,929 (56.95%)	0.00

Data are n or n (%).

a Includes individuals of other ethnicities or mixed ethnicities.

b Standardised-mean-difference, SMD after overlap weighting of dengue cases and population-based controls without dengue, weighted from original samples.

c Comorbidity burden was defined using the Charlson Comorbidity Index (CCMI), which consists of the following comorbidities: myocardial infarction, chronic heart failure, peripheral vascular disease, cerebrovascular accident, dementia, chronic obstructive pulmonary disease, connective tissue disease, peptic ulcer disease, diabetes mellitus, hemiplegia, liver disease, moderate to severe renal impairment, solid tumor, leukemia, human immunodeficiency virus (HIV) infection with AIDS.

**Fig. 2:**
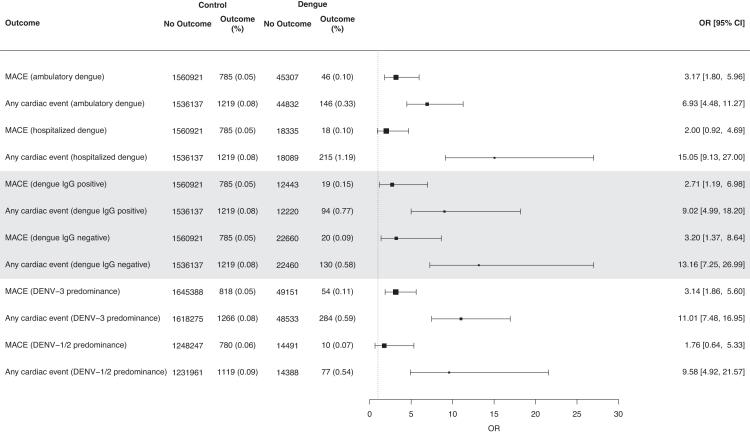
Forest plot of odds ratios (ORs) for new-onset acute cardiac events in dengue cases stratified by IgG positivity, predominant DENV-serotype, and severity, versus population-based controls without dengue. OR> 1 (dotted-line) denotes higher odds of a respective composite/individual outcome amongst dengue cases and population-based controls without dengue. ORs were estimated using logistic regression, with overlap weights applied; weights were estimated based on demographic characteristics (age, sex, ethnicity), socioeconomic status (housing type), comorbidities, and healthcare utilisation. Error bars represent 95% confidence-intervals for estimates of ORs. Major-adverse-cardiac-event (MACE) was defined as the first incidence of myocardial infarction, stroke, ventricular arrhythmia, or sudden cardiac death. Any acute cardiac event was taken as a composite of any ischemic heart disease, dysrhythmias, inflammatory heart disease, other cardiac disease, and thrombotic conditions.

Elevated odds of any new-incident cardiovascular event (adjusted-odds-ratio, aOR = 10.63 [95% CI = 7.56, 15.48]), major-adverse-cardiac-event (MACE) (aOR = 2.92 [95% CI = 1.81–4.88]), ischemic-heart-disease (aOR = 3.00 [95% CI = 1.83–5.15]), and dysrhythmia (aOR = 18.44 [95% CI = 11.25 = 32.96]) were observed up to 30 days post-DENV-infection, versus uninfected controls (Table 2). Of the 271 new-onset dysrhythmia events recorded following acute dengue infection, atrial fibrillation/flutter comprised the majority of events (49.8%, 135/271). However, overall excess burden (EB) of cardiovascular events in DENV-infected adults versus population-based controls was modest, with <1 excess event per-100-persons over follow-up time, across all specified outcomes (Table 2). Odds of acute cardiovascular events remained elevated in both ambulatory/hospitalised DENV-infected cases (ambulatory: aOR = 6.93 [95% CI = 4.48–11.27]; hospitalised: aOR = 15.05 [95% CI = 9.13–27.00]), DENV-IgG-positive/negative cases (DENV-IgG positive: aOR = 9.02 [95% CI = 4.99–18.20]); DENV-IgG negative: aOR = 13.16 [95% CI = 7.25–26.99]), and across DENV1/2 and DENV3-predominant transmission (DENV1/2: aOR = 9.58 [95% CI = 4.92–21.57]; DENV3: aOR = 11.01 [95% CI = 7.48–16.95]), versus uninfected controls (Fig. 2, Supplementary Table S1). <1% (361/62,921) of DENV-infected cases without prior cardiac history reported a new-onset cardiac event within 30-days. 1.2% (215/18,089) of hospitalised DENV-infected cases without prior cardiac history reported a new-onset cardiac event, versus 0.33% (146/44832) of DENV-infected cases that did not require hospitalisation for dengue.

**Table 2:** Odds and excess burden of new-onset acute cardiac events in dengue cases, versus population-based controls without dengue.

Outcomes	Adjusted odds-ratio, aOR,^a^ 95% CI	p-value (aOR)	Excess burden (EB) per-100-persons, 95% CI	Controls without dengue (N)	Controls without dengue, with outcome N (%)	Dengue cases (N)	Dengue cases with outcome N (%)
**All dengue cases versus population-based controls without dengue**							
**Composite acute cardiac events**							
Any major-adverse-cardiac-event (MACE)^b^	2.92 (1.81, 4.88)	<0.001	0.07 (0.04, 0.10)	1,560,921	785 (0.05)	63,642	64 (0.10)
Any acute cardiac event^c^	10.63 (7.56, 15.48)	<0.001	0.53 (0.47, 0.60)	1,536,137	1219 (0.08)	62,921	361 (0.57)
**Acute cardiac events**^d^							
Dysrhythmia^e^	18.44 (11.25, 32.96)	<0.001	0.41 (0.35, 0.46)	1,586,695	536 (0.03)	64,306	271 (0.42)
Ischemic heart disease	3.00 (1.83, 5.15)	<0.001	0.06 (0.04, 0.09)	1,565,431	710 (0.05)	63,753	60 (0.09)

OR> 1 denotes higher odds of a respective composite/individual outcome amongst dengue cases and population-based controls without dengue.

Abbreviations: CI, confidence interval; OR, odds ratio; EB, excess burden.

a Logistic regression, with overlap weights applied; weights were estimated based on demographic characteristics (age, sex, ethnicity), socioeconomic status (housing type), comorbidities, and healthcare utilisation. EBs were computed by taking the differences in weighted incidences between comparator groups.

b Major-adverse-cardiac-event (MACE) was defined as the first incidence of myocardial infarction, stroke, ventricular arrhythmia, or sudden cardiac death.

c Any acute cardiac event was taken as a composite of any ischemic heart disease, dysrhythmias, inflammatory heart disease, other cardiac disease, and thrombotic conditions.

d Although cases of new-incident heart disease (eg. myocarditis/pericarditis), other cardiac disease (eg. cardiomyopathy/cardiogenic shock) and thrombotic conditions (eg. deep venous thrombosis) were included in the composite of acute cardiac events, separate categories were not computed given the small number of incident cases.

e Of the 271 new-onset dysrhythmia events recorded following acute dengue infection, atrial fibrillation/flutter comprised the majority of events (49.8%, 135/271); other dysrhythmias included sinus bradycardia (N = 74), sinus tachycardia (N = 54), and other arrhythmias (N = 8).

**Fig. 3:**
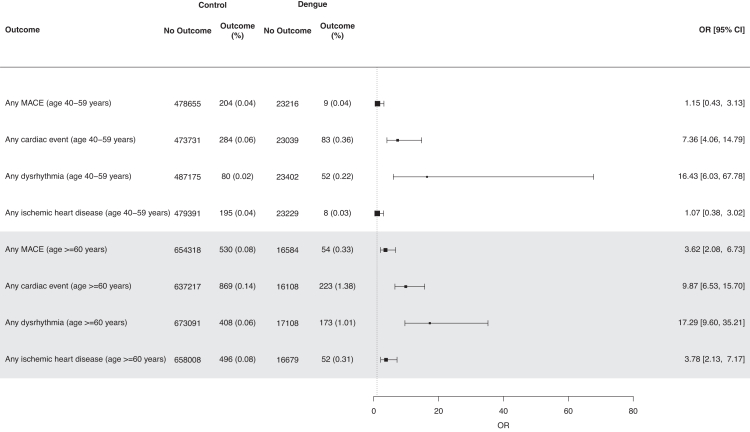
Forest plot of odds ratios (ORs) for new-onset acute cardiac events in dengue cases, versus population-based controls without dengue (age subgroups). OR> 1 (dotted-line) denotes higher odds of a respective composite/individual outcome amongst dengue cases and population-based controls without dengue. ORs were estimated using logistic regression, with overlap weights applied; weights were estimated based on demographic characteristics (age, sex, ethnicity), socioeconomic status (housing type), comorbidities, and healthcare utilisation. Error bars represent 95% confidence-intervals for estimates of ORs. Major-adverse-cardiac-event (MACE) was defined as the first incidence of myocardial infarction, stroke, ventricular arrhythmia, or sudden cardiac death. Any cardiac event was taken as a composite of any ischemic heart disease, dysrhythmias, inflammatory heart disease, other cardiac disease, and thrombotic conditions. The age 18–39 subgroup was excluded as the relatively lower risk of acute cardiac events in young adults meant that estimation of ORs for several of the cardiac outcomes was not possible, due to zero events in some categories.

In subgroup analyses by age, consistently elevated odds of any new-incident cardiovascular event, MACE, dysrhythmia and ischemic-heart-disease was still observed up to 30 days post-DENV-infection, versus uninfected controls (Fig. 3); with the exception of MACE (aOR = 1.15 [95% CI = 0.43–3.13]) and ischemic-heart-disease (aOR = 1.07 [95% CI = 0.38–3.02]) in those aged 40–59 years. However, increased odds of any acute cardiovascular event (aOR = 7.36 [95% CI = 4.06–14.79]) was still observed in DENV-infected individuals versus uninfected controls, aged 40–59 years (Fig. 3, Supplementary Table S2). Overall EB of acute cardiovascular events in DENV-infected adults was however modest, with <1 excess event per-100-persons across all specified outcomes (Supplementary Table S2), except amongst those aged ≥60 years (EB = 1.25 [95% CI = 1.05–1.44]).

In sensitivity analyses, odds of any cardiovascular event (aOR = 9.96 [95% CI = 6.98–14.67]), MACE (aOR = 2.04 [95% CI = 1.15–3.66]), ischemic-heart-disease (aOR = 2.07 [95% CI = 1.14–3.82]), and dysrhythmias (aOR = 17.50 [95% CI = 10.53–31.57]) remained significantly elevated up to 30 days in NS-1 positive DENV-infected cases, versus population-based controls without DENV-infection (Supplementary Table S3). Significantly elevated risk of AMI was still observed in DENV-infected cases, versus uninfected population-based controls, regardless of whether registry records (SMIR) or healthcare-claims data were utilised to define AMI events (registry records: aOR = 6.50 [95% CI = 2.89–17.76]; claims data: aOR = 9.93 [95% CI = 4.47–27.43]) (Supplementary Table S4). When Cox regression was utilised to evaluate the hazard-ratio of new-onset cardiovascular events, significantly elevated risk (adjusted hazard-ratio, aHR) for MACE (aHR = 2.86 [95% CI = 2.21–3.72]), dysrhythmia (aHR = 18.31 [95% CI = 15.64–21.44]) and ischemic-heart-disease (aHR = 2.99 [95% CI = 2.29–3.92] was still observed in DENV-infected cases, versus uninfected population-based controls (Supplementary Table S5). Usage of alternative weighting schemes (inverse-probability weights) or a doubly-robust approach incorporating covariates together with inverse probability weights employed in the outcome regression did not significantly skew risk estimates (Supplementary Table S6). Risks of acute cardiovascular events in DENV-infected individuals (N = 62,346) were contrasted against that following SARS-CoV-2-infection (N = 1,440,779); differences in baseline characteristics post-weighting were minimal with SMDs <0.05 (Supplementary Table S7). Odds of MACE (aOR = 2.29 [95% CI = 1.46–3.70]), any cardiovascular event (aOR = 4.70 [95% CI = 3.65–6.14]), ischemic-heart-disease (aOR = 2.27 [95% CI = 1.43–3.71]) and dysrhythmias (aOR = 5.47 [95% CI = 4.02–7.60]) were consistently elevated post-DENV infection, versus COVID-19 (Supplementary Table S8). Odds of bronchitis, the negative-outcome control, were not significantly different (aOR = 0.64 [95% CI = 0.03–9.11]) in the 0–30 days post-DENV infection, versus uninfected controls (Supplementary Table S9). The E-value for association between DENV-infection and any acute cardiac event was 20.75; it was deemed unlikely that an unmeasured or unknown confounder would have a relative risk exceeding the E-value, given that ORs for known risk factors associated with acute cardiac events (eg. older age, gender, non-Chinese ethnicity) ranged between 1 and 4 in our study cohort on regression analysis.

## Discussion

In a national cohort of adult Singaporeans (N = 65,207) with DENV-infection, odds of any cardiovascular event, MACE, ischemic-heart-disease, and dysrhythmias were significantly elevated up to 30 days following acute DENV-infection, when contrasted against population-based controls without DENV-infection. Overall excess burden (EB) of acute cardiovascular events in DENV-infected adults was modest, with <1 excess event per-100-cases except amongst those aged ≥60 years. Higher odds of an acute cardiac event were observed following DENV-infection, versus other viral infections, specifically COVID-19.

Cardiovascular involvement, including myocarditis and pericarditis, has been anecdotally documented as a rare feature of DENV-infection,^2^^,^^3^ and increased risk of MACE in the acute 30-day period following dengue infection has been observed in population-based studies.^8^^,^^9^ However, evidence for a broader spectrum of cardiac events following acute dengue infection, including dysrhythmias and ischemic-heart-disease, is still emerging. In animal models, both primary and secondary DENV-infection were shown to induce marked cardiac dysfunction and cardiac tissue damage,^20^^,^^21^ which was associated with inflammation and electrophysiological changes in the heart.^21^ Evidence for cardiac rhythm abnormalities during the convalescent stage of acute DENV-infection, however, was previously limited to small prospective cohorts and case reports of non-resolving atrial fibrillation following DENV-infection.^4^^,^^22^^,^^23^ In a small prospective pediatric cohort of DENV-infected patients (N = 35) followed prospectively with 24-h Holter monitoring, cardiac rhythm abnormalities were detected in a third of patients during the convalescent phase.^22^ In our large population-based cohort, increased odds of dysrhythmia post-DENV infection were consistently observed across both DENV1-2 and DENV3-predominant transmission, in possible secondary dengue (DENV-IgG-positive) as well as IgG-negative cases, and in hospitalised/non-hospitalised DENV-infected cases, versus uninfected controls. DENV-induced arrhythmias are postulated to result from myocardial oedema and electrical disturbances arising from immune-mediated damage and disrupted calcium regulation in cardiomyocytes.^2^ In a systematic review of acute cardiovascular sequelae post-DENV infection that included ∼7000 patients, the majority of reported cardiac manifestations included electrocardiogram (ECG) abnormalities, such as sinus bradycardia and ST-changes.^3^ ECG abnormalities were, in turn, associated with increased risk of adverse cardiac outcomes in a small prospective cohort (N = 320) of hospitalised DENV-infected patients.^6^

Early identification of cardiovascular involvement in DENV-infected individuals may be useful in prognostication. In a multi-centre cohort that included hospitalised cases of severe DENV-infection, significantly higher rates of early mortality were observed in severe DENV-infected patients with cardiac involvement (elevated troponin).^24^ Similarly, in a Brazilian cohort that self-reported prior DENV-infection and underwent echocardiography, a history of self-reported DENV-infection was significantly associated with myocardial alterations including lower left-ventricular-ejection-fraction (LVEF) and reduced global-longitudinal-strain (GLS), which could be detected by conventional transthoracic echocardiography.^25^ Close follow-up of individuals with cardiovascular involvement in the acute phase of DENV-infection is warranted to assess for subsequent resolution, given concern for long-term cardiovascular sequelae following DENV-infection.^13^^,^^14^ In a retrospective population-based cohort study, over the post-acute period (31–300 days post-infection), a 46.0% increase in risk of cardiovascular sequelae in DENV-infected individuals versus uninfected controls was observed, with elevated risk of MACE, dysrhythmia and ischemic-heart-disease in the post-acute phase.^14^ Higher risk of post-acute cardiovascular sequelae following DENV-infection was also observed in comparison with other viral infections, specifically COVID-19.^13^ Elevated risk of cardiovascular events post-DENV-infection is of significance, given the significant contribution of cardiac events to mortality post-DENV-related hospitalisation,^26^ and resurgence of DENV-infection due to global warming and climate change, resulting in autochthonous dengue transmission occurring even within temperate regions during favourable seasons.^7^

Cardiovascular risk in acute DENV-infection, however, needs to be contextualised in the setting of low overall incidence in the population at-large. <1% (361/62,921) of DENV-infected cases without prior cardiac history reported a new-onset cardiac event within 30-days, and EB was <1 excess event per-100-persons for the majority of outcomes post-DENV-infection, versus population-based controls. In contrast, prior estimates of acute cardiac involvement in DENV-infection ranged from 15 to 43.6%, albeit from small prospective cohorts predominantly comprised of patients hospitalised for acute DENV-infection,^4^, ^5^, ^6^ with short duration (median: 4 days) of dengue-associated symptoms prior to admission.^4^ Surveillance and assessment of potential cardiovascular complications in patients recovering from acute DENV-infection should therefore be focused on populations at highest risk, specifically in hospitalised cases and older adults with potential pre-existing comorbidities or cardiovascular risk factors; given significant challenges in the implementation of cardiovascular imaging and screening for DENV-infected individuals in under-resourced tropical settings.^27^ Examples of such challenges include low awareness of cardiovascular complications in DENV-infection, limited access to technology including cardiac imaging, and lack of trained personnel.^27^ Various alternatives, such as usage of portable cardiac point-of-care ultrasound (POCUS) and tele-echocardiography in remote areas,^27^ together with rapid cardiovascular-magnetic-resonance (CMR) protocols to reduce cost and improve accessibility in LMICs,^28^ could potentially enhance cardiovascular care following DENV-infection, though additional longitudinal studies linking early imaging alterations with long-term cardiovascular outcomes are required. Higher EB of acute cardiovascular events in older adults likely reflects the vulnerability of elderly dengue survivors and those with increased comorbidity burden, versus younger counterparts.^29^ Additional studies are required to evaluate if early detection and better management of cardiovascular complications in the acute phase translates into lower risk of post-acute cardiovascular sequelae in at-risk groups, given that elevated risk of post-acute cardiovascular sequelae 31–300 days post-DENV-infection similarly accrued in older individuals and those with comorbidities.^13^^,^^14^

The present study has several strengths. Comprehensive nationwide registries were utilised to classify DENV-infection, with supporting diagnostic confirmation; reducing risk of misclassification. Healthcare-claims databases with national-level coverage were utilised to determine occurrence of cardiovascular events, minimising loss-to-follow-up bias. Cardiovascular sequelae post-DENV-infection were contrasted against uninfected controls, allowing population-based estimates of risks/EB. However, limitations are as follows. Comparisons of risks for cardiovascular sequelae post-DENV-infection, versus COVID-19 cases, were exploratory in nature. Cardiovascular risks following DENV-infection may differ substantially from other non-SARS-CoV-2 viral infections, though extensive surveillance/testing databases were only available for DENV/COVID-19 in our local context. In this observational study, correlation does not imply causation; additionally, it was not possible to distinguish if DENV-infection directly resulted in an acute cardiac event, or if the physiological stress of acute infection unmasked an already underlying predisposition. A proportion of DENV-infections may be asymptomatic and hence unrecognised/under-reported^30^; biasing estimates of risk in a conservative direction. Lack of information on DENV-IgG titre and absence of paired serological sampling meant that secondary dengue cases could be potentially misclassified. DENV serotype was imputed based on prevailing serotype from virological surveillance and not individual-level sequencing, resulting in potential misclassification. Healthcare-claims data might under-report estimates of milder cardiovascular sequelae not affecting reimbursement. While significantly elevated AMI risk was still observed in DENV-infected cases versus uninfected population-based controls, regardless of whether AMI events were registry-based or identified using healthcare-claims data, similar comparative analyses could not be performed for other cardiac events where no equivalent national registry existed (eg. dysrhythmias). Additionally, it was not possible to assess if complications (eg. dysrhythmias) persisted over time or subsequently resolved. Individuals with missing data were excluded from analysis; however, as the proportion of individuals with missing data was miniscule due to usage of national electronic-health-records/databases, this limited potential bias introduced by the exclusion of such individuals. While baseline sociodemographic and clinical characteristics were adjusted for, unmeasured confounders could introduce potential bias. For instance, physical measurements (eg. body-mass-index) were unavailable in national electronic-health-records and hence obesity, a key cardiovascular risk factor, could not be adjusted for. As subgroup analyses conducted reduced the number of observable events and sample size, the precision of estimates may be lower versus primary analyses. Finally, though this study was conducted in a multi-ethnic cohort, findings may not be generalisable to other populations, especially those of non-Asian ethnicity, and pediatric populations; future exploratory analyses are necessary to evaluate cardiovascular risk following DENV-infection in these specific populations.

In conclusion, acute DENV-infection was associated with significantly higher odds of new-incident cardiovascular events up to 30 days post-infection, compared with uninfected population-based controls, though excess-burden was modest. Older individuals at higher risk should be monitored for cardiovascular complications following DENV-infection.

## Contributors

LEW, CJY and JTL contributed to literature search and writing of the manuscript. TWZ, CC, LCN, PYC, YSL, MRBA, JY, KKY, MCYY, DH, DCL and KBT contributed to critical review and editing of the manuscript. DCL and KBT provided supervision. KBT, LEW, JTL contributed to study design. TWZ, CJY contributed to data collection, and TWZ contributed to data analysis. All authors had full access to all the data in the study and take responsibility for the decision to submit for publication. LEW, JTL and TWZ directly accessed and verified the underlying data reported in the manuscript. The corresponding author is the guarantor and accepts full responsibility for the work and/or the conduct of the study, had access to the data, and controlled the decision to publish. The corresponding author attests that all listed authors meet authorship criteria and that no others meeting the criteria have been omitted.

## Data sharing statement

The databases with individual-level information used for this study are not publicly available due to personal data protection. Deidentified data can be made available for research, subject to approval by the Ministry of Health of Singapore. All inquiries should be sent to the corresponding author.

## Declaration of interests

All authors have completed the ICMJE uniform disclosure form and declare: no support from any organisation for the submitted work; no financial relationships with any organisations that might have an interest in the submitted work in the previous three years; no other relationships or activities that could appear to have influenced the submitted work.

## References

[1] GBD 2021 Forecasting Collaborators (2024). Burden of disease scenarios for 204 countries and territories, 2022-2050: a forecasting analysis for the global burden of disease study 2021. Lancet.

[2] Yacoub S., Wertheim H., Simmons C.P., Screaton G., Wills B. (2014). Cardiovascular manifestations of the emerging dengue pandemic. Nat Rev Cardiol.

[3] (2022). Cardiovascular sequelae of dengue fever: a systematic review. Expert Rev Cardiovasc Ther.

[4] Miranda C.H., Borges Mde C., Matsuno A.K. (2013). Evaluation of cardiac involvement during dengue viral infection. Clin Infect Dis.

[5] Shah C., Vijayaraghavan G., Kartha C.C. (2021). Spectrum of cardiac involvement in patients with dengue fever. Int J Cardiol.

[6] Mansanguan C., Hanboonkunupakarn B., Muangnoicharoen S. (2021). Cardiac evaluation in adults with dengue virus infection by serial echocardiography. BMC Infect Dis.

[7] Jacobsen A.P., Khiew Y.C., Duffy E. (2022). Climate change and the prevention of cardiovascular disease. Am J Prev Cardiol.

[8] Wei K.C., Sy C.L., Wang W.H., Wu C.L., Chang S.H., Huang Y.T. (2022). Major acute cardiovascular events after dengue infection-A population-based observational study. PLoS Negl Trop Dis.

[9] Wei K.C., Wang W.H., Wu C.L., Chang S.H., Huang Y.T. (2023). Heart failure after dengue infection- a population-based self-controlled case-series study. Travel Med Infect Dis.

[10] Tang N., Lim J.T., Dickens B. (2024). Effects of recent prior dengue infection on risk and severity of subsequent SARS-CoV-2 infection: a retrospective cohort study. Open Forum Infect Dis.

[11] Tan C.C., Lam C.S.P., Matchar D.B., Zee Y.K., Wong J.E.L. (2021). Singapore's health-care system: key features, challenges, and shifts. Lancet.

[12] Wee L.E., Malek M.I.B.A., Tan J. (2024). Risk of death and cardiovascular events following COVID-19 vaccination or positive SARS-CoV-2 test amongst adult Singaporeans during omicron transmission. Vaccine.

[13] Wee L.E., Lim J.T., Tan J.Y.J. (2024). Dengue versus COVID-19: comparing the incidence of cardiovascular, neuropsychiatric and autoimmune complications. J Travel Med.

[14] Lim J.T., Wee L.E., Tan W.Z. (2025). Characterization of post-acute multi-organ sequelae following dengue infection. Clin Microbiol Infect.

[15] Chew N.W.S., Chong B., Kuo S.M. (2023). Trends and predictions of metabolic risk factors for acute myocardial infarction: findings from a multiethnic nationwide cohort. Lancet Reg Health West Pac.

[16] Li F., Thomas L.E., Li F. (2019). Addressing extreme propensity scores via the overlap weights. Am J Epidemiol.

[17] Pillay K., Keddie S.H., Fitchett E. (2025). Evaluating the performance of common reference laboratory tests for acute dengue diagnosis: a systematic review and meta-analysis of RT-PCR, NS1 ELISA, and IgM ELISA. Lancet Microbe.

[18] Tan R.Y., Wong B., Lim R. (2023). Factors associated with delayed diagnosis of symptomatic adult COVID-19 cases presenting to primary care: a population-wide study during transition from Delta to omicron BA.1 in Singapore. Lancet Reg Health West Pac.

[19] Haneuse S., VanderWeele T.J., Arterburn D. (2019). Using the E-Value to assess the potential effect of unmeasured confounding in observational studies. JAMA.

[20] Jácome F.C., Teixeira A.L., Coutinho D.D. (2019). Secondary dengue infection in immunocompetent murine model leads to heart tissue damage. Acta Virol.

[21] Kangussu L.M., Costa V.V., Olivon V.C. (2022). Dengue virus infection induces inflammation and oxidative stress on the heart. Heart.

[22] La-Orkhun V., Supachokchaiwattana P., Lertsapcharoen P., Khongphatthanayothin A. (2011). Spectrum of cardiac rhythm abnormalities and heart rate variability during the convalescent stage of dengue virus infection: a holter study. Ann Trop Paediatr.

[23] Mahmod M., Darul N.D., Mokhtar I., Nor N.M., Anshar F.M., Maskon O. (2009). Atrial fibrillation as a complication of dengue hemorrhagic fever: non-self-limiting manifestation. Int J Infect Dis.

[24] Lee I.K., Chen Y.H., Huang C.H. (2022). A multicenter cohort study of severe dengue and critically ill influenza patients with elevated cardiac troponin-I: difference clinical features and high mortality. Travel Med Infect Dis.

[25] Kaagaard M.D., Wegener A., Gomes L.C. (2022). Potential role of transthoracic echocardiography for screening LV systolic dysfunction in patients with a history of dengue infection. A cross-sectional and cohort study and review of the literature. PLoS One.

[26] Lee J.C., Cia C.T., Lee N.Y., Ko N.Y., Chen P.L., Ko W.C. (2022). Causes of death among dengue patients causes of death among hospitalized adults with dengue fever in Tainan, 2015: emphasis on cardiac events and bacterial infections. J Microbiol Immunol Infect.

[27] Barberato S.H., Cardim N., Rassi D.D.C. (2025). Cardiac imaging in patients with tropical diseases-a scientific statement of the European Association of Cardiovascular Imaging (EACVI) of the European Society of Cardiology and the Cardiovascular Imaging Department of the Brazilian Society of Cardiology (DICSBC). Eur Heart J Cardiovasc Imaging.

[28] Menacho K.D., Ramirez S., Perez A. (2022). Improving cardiovascular magnetic resonance access in low- and middle-income countries for cardiomyopathy assessment: rapid cardiovascular magnetic resonance. Eur Heart J.

[29] Lin R.J., Lee T.H., Leo Y.S. (2017). Dengue in the elderly: a review. Expert Rev Anti Infect Ther.

[30] Yap G., Li C., Mutalib A., Lai Y.L., Ng L.C. (2013). High rates of inapparent dengue in older adults in Singapore. Am J Trop Med Hyg.

