# Supplementary material for: Cardiovascular complications in acute dengue infection: a population-based cohort study
Source: Lancet Reg Health West Pac. 2025 Oct 16;64:101713. doi: 10.1016/j.lanwpc.2025.101713 (PMC12554185; doi:10.1016/j.lanwpc.2025.101713)
Supplement: Supplementary Figure and Tables [file mmc1.docx]

**Supplementary Material, Table of Contents**

- Supplementary Table 1: Odds and excess burden of new-onset acute cardiac events in dengue cases stratified by IgG positivity, predominant DENV-serotype in circulation, and severity (hospitalised/ambulatory), versus population-based controls without dengue (pg. 2-3)
- Supplementary Table 2: Odds and excess burden of new-onset acute cardiac events in dengue cases, versus population-based controls without dengue (age subgroups) (pg. 4)
- Supplementary Table 3: Odds and excess burden of new-onset acute cardiac events in NS1-positive dengue cases, versus population-based controls without dengue (pg. 5)
- Supplementary Table 4: Odds and excess burden of new-onset acute myocardial infarction in dengue cases, versus population-based controls without dengue (from 1st Jan 2017-31st Dec 2021), using a)national healthcare claims data; b)acute myocardial infarction registry data (pg.6)
- Supplementary Table 5: Hazards-ratio and excess burden of new-onset acute cardiac events in dengue cases, versus population-based controls without dengue (pg. 7-9)
- Supplementary Table 6: Odds of acute cardiac events in dengue cases versus population-based controls without dengue, using alternative weighting schemes (pg. 10)
- Supplementary Table 7: Baseline characteristics of Singaporean adults with dengue or COVID-19, with standardised-mean-differences before and after overlap weighting

(pg. 11,12)

- Supplementary Table 8: Odds of new-onset acute cardiac events in dengue cases versus COVID-19 cases (pg. 13)
- Supplementary Table 9: Odds of negative outcome control (bronchitis) in dengue cases versus population-based controls without dengue (pg.14)
- Supplementary Figure 1: Cohort construction flowchart (dengue versus COVID-19) (pg. 15)
- List of ICD-10 codes used for cardiovascular diagnoses of interest (pg 16-19)

**Supplementary Table 1: Odds and excess burden of new-onset acute cardiac events in dengue cases stratified by IgG positivity, predominant DENV-serotype in circulation, and severity (hospitalised/ambulatory), versus population-based controls without dengue**

| **Outcomes** | **Adjusted odds-ratio, aOR,^a^**  **95% CI** | **p-value (aOR)** | **Excess burden (EB) per-100-persons, 95%CI** | **Controls without dengue (N)** | **Controls without dengue,  with outcome N(%)** | **Dengue cases (N)** | **Dengue cases  with outcome N(%)** |
| --- | --- | --- | --- | --- | --- | --- | --- |
| **Ambulatory dengue cases versus population-based controls without dengue^e^** | | | | | | | |
| **Composite acute cardiac events** |  |  |  |  |  |  |  |
| Any major-adverse-cardiac-event (MACE)^b^ | **3.17 (1.80,5.96)** | **<0.001** | 0.07 (0.04,0.11) | 1560921 | 785 (0.05) | 45307 | 46 (0.10) |
| Any acute cardiac event^c^ | **6.93 (4.48,11.27)** | **<0.001** | 0.29 (0.23,0.35) | 1536137 | 1219 (0.08) | 44832 | 146 (0.33) |
| **Acute cardiac events^d^** |  |  |  |  |  |  |  |
| Dysrhythmia | **11.94 (6.26,26.27)** | **<0.001** | 0.21 (0.16,0.26) | 1586695 | 536 (0.03) | 45681 | 100 (0.22) |
| Ischemic heart disease | **3.24 (1.79,6.26)** | **<0.001** | 0.07 (0.03,0.1) | 1565431 | 710 (0.05) | 45378 | 43 (0.10) |
| **Hospitalised dengue cases versus population-based controls without dengue^e^** | | | | | | | |
| **Composite acute cardiac events** |  |  |  |  |  |  |  |
| Any major-adverse-cardiac-event (MACE)^b^ | 2.00(0.92,4.69) | 0.091 | 0.05 (-0.01,0.11) | 1560921 | 785 (0.05) | 18335 | 18 (0.10) |
| Any acute cardiac event^c^ | **15.05 (9.13,27.00)** | **<0.001** | 1.11 (0.94,1.27) | 1536137 | 1219 (0.08) | 18089 | 215 (1.19) |
| **Acute cardiac events^d^** |  |  |  |  |  |  |  |
| Dysrhythmia | **27.46 (13.42,68.82)** | **<0.001** | 0.88 (0.74,1.03) | 1586695 | 536 (0.03) | 18625 | 171 (0.92) |
| Ischemic heart disease | 2.04 (0.91,4.93) | 0.094 | 0.05 (-0.01,0.10) | 1565431 | 710 (0.05) | 18375 | 17 (0.09) |
| **Possible secondary dengue cases (IgG positive), versus population-based controls without dengue^f^** | | | | | | | |
| **Composite acute cardiac events** |  |  |  |  |  |  |  |
| Any major-adverse-cardiac-event (MACE)^b^ | **2.71 (1.19-6.98)** | **0.020** | 0.10 (0.02-0.18) | 1560921 | 785 (0.05) | 12443 | 19 (0.15) |
| Any acute cardiac event^c^ | **9.02 (4.99-18.20)** | **<0.001** | 0.68 (0.52-0.85) | 1536137 | 1219 (0.08) | 12220 | 94 (0.77) |
| **Acute cardiac events^d^** |  |  |  |  |  |  |  |
| Dysrhythmia | **14.74 (6.37-44.63)** | **<0.001** | 0.49 (0.36-0.63) | 1586695 | 536 (0.03) | 12631 | 67 (0.53) |
| Ischemic heart disease | **3.04 (1.29-8.23)** | **0.020** | 0.10 (0.02-0.18) | 1565431 | 710 (0.05) | 12484 | 19 (0.15) |
| **Possible primary dengue cases (IgG negative), versus population-based controls without dengue^f^** | | | | | | | |
| **Composite acute cardiac events** |  |  |  |  |  |  |  |
| Any major-adverse-cardiac-event (MACE)^b^ | **3.20 (1.37-8.64)** | **0.011** | 0.06 (0.02-0.11) | 1560921 | 785 (0.05) | 22660 | 20 (0.09) |
| Any acute cardiac event^c^ | **13.16 (7.25-26.99)** | **<0.001** | 0.54 (0.44-0.65) | 1536137 | 1219 (0.08) | 22460 | 130 (0.58) |
| **Acute cardiac events^d^** |  |  |  |  |  |  |  |
| Dysrhythmia | **21.21 (9.09-66.19)** | **<0.001** | 0.39 (0.30-0.47) | 1586695 | 536 (0.03) | 22818 | 92 (0.40) |
| Ischemic heart disease | **3.16 (1.30-8.96)** | **0.021** | 0.06 (0.01-0.10) | 1565431 | 710 (0.05) | 22694 | 18 (0.08) |
| **Dengue cases versus population-based controls without dengue, DENV3-predominant transmission^g^** | | | | | | | |
| **Composite acute cardiac events** |  |  |  |  |  |  |  |
| Any major-adverse-cardiac-event (MACE)^b^ | **3.14 (1.86,5.60)** | **<0.001** | 0.08 (0.04,0.11) | 1645388 | 818 (0.05) | 49151 | 54 (0.11) |
| Any acute cardiac event^c^ | **11.01 (7.48,16.95)** | **<0.001** | 0.54 (0.47,0.61) | 1618275 | 1266 (0.08) | 48533 | 284 (0.59) |
| **Acute cardiac events^d^** |  |  |  |  |  |  |  |
| Dysrhythmia | **20.84 (11.52,42.88)** | **<0.001** | 0.40 (0.34,0.45) | 1673271 | 512 (0.03) | 49693 | 203 (0.41) |
| Ischemic heart disease | **3.10 (1.80,5.65)** | **<0.001** | 0.07 (0.04,0.10) | 1650133 | 764 (0.05) | 49244 | 50 (0.10) |
| **Dengue cases versus population-based controls without dengue, DENV1/2-predominant transmission^g^** | | | | | | | |
| **Composite acute cardiac events** |  |  |  |  |  |  |  |
| Any major-adverse-cardiac-event (MACE)^b^ | 1.76 (0.64,5.33) | 0.284 | 0.03 (-0.02,0.08) | 1248247 | 780 (0.06) | 14491 | 10 (0.07) |
| Any acute cardiac event^c^ | **9.58 (4.92,21.57)** | **<0.001** | 0.48 (0.36,0.61) | 1231961 | 1119 (0.09) | 14388 | 77 (0.54) |
| **Acute cardiac events^d^** |  |  |  |  |  |  |  |
| Dysrhythmia | **23.48 (8.59,99.55)** | **<0.001** | 0.45 (0.34,0.57) | 1265720 | 453 (0.04) | 14613 | 68 (0.46) |
| Ischemic heart disease | 1.88 (0.68,5.88) | 0.241 | 0.03 (-0.02,0.09) | 1251760 | 709 (0.06) | 14509 | 10 (0.07) |

OR> 1 denotes higher odds of a respective composite/individual outcome amongst dengue cases and population-based controls without dengue

Abbreviations: CI, confidence interval; OR, odds ratio; EB, excess burden

^a^ Logistic regression, with overlap weights applied; weights were estimated based on demographic characteristics (age, sex, ethnicity), socioeconomic status (housing type), comorbidities, and healthcare utilisation. EBs were computed by taking the differences in weighted incidences between comparator groups.

^b^ Major-adverse-cardiac-event (MACE) was defined as the first incidence of myocardial infarction, stroke, ventricular arrhythmia, or sudden cardiac death.

^c^ Any acute cardiac event was taken as a composite of any ischemic heart disease, dysrhythmias, inflammatory heart disease, other cardiac disease, and thrombotic conditions.

^d^ Although cases of new-incident inflammatory heart disease (eg. myocarditis/pericarditis), other cardiac disease (eg. cardiomyopathy/cardiogenic shock) and thrombotic conditions (eg. deep venous thrombosis) were included in the composite of acute cardiac events, separate categories were not computed given the small number of incident cases

^e^ Hospitalisations attributed to dengue (ICD-10 code A90/A91) occurring within 30 days of a positive dengue test were categorised as dengue hospitalisations; reported dengue cases without a dengue-related hospitalisation were classified as ambulatory cases.

^f^ Acute dengue cases (NS1/IgM positive) with concurrent positive IgG were classified as possible secondary dengue cases; acute dengue cases (NS1/IgM positive) with concurrent negative dengue IgG were classified as possible primary dengue cases. Numbers do not add up to the original number of dengue cases because acute dengue cases (NS1/IgM positive) that were not concurrently tested for dengue IgG were excluded.

^g^ Dengue cases recorded before January 2020 were attributed to DENV1/2; dengue cases recorded after January 2020 onwards were attributed to DENV3. In January 2020, surveillance detected a shift from DENV1/2 to DENV-3, which coincided with a surge in dengue infections; Singapore had not experienced a DENV3 outbreak in the past 30 years. The total number of uninfected population controls differs from the original number in the main cohort (N=1616865) because in the main cohort, T_0_ in uninfected population controls was assigned from a pool of dates ranging from 2017-2023 (matching distribution of the dengue cases); whereas T_0_ for uninfected population controls was assigned from a pool of pre-2020 dates (for comparison against DENV1/2 cases) and for post-2020 dates (for comparison against DENV3 cases), respectively.

**Supplementary Table 2:** **Odds and excess burden of new-onset acute cardiac events in dengue cases, versus population-based controls without dengue (age subgroups)**

| **Outcomes** | **Adjusted odds-ratio, aOR,^a^**  **95% CI** | **p-value (aOR)** | **Excess burden (EB) per-100-persons, 95%CI** | **Controls without dengue (N)^e^** | **Controls without dengue,  with outcome N(%)** | **Dengue cases (N)** | **Dengue cases  with outcome N(%)** |
| --- | --- | --- | --- | --- | --- | --- | --- |
| **All dengue cases versus population-based controls without dengue, aged 40-59 years^e^** | | | | | | | |
| **Composite acute cardiac events** |  |  |  |  |  |  |  |
| Any major-adverse-cardiac-event (MACE)^b^ | 1.15 (0.43,3.13) | 0.786 | 0.01 (-0.03,0.04) | 478655 | 204 (0.04) | 23216 | 9 (0.04) |
| Any acute cardiac event^c^ | **7.36 (4.06,14.79)** | **<0.001** | 0.32 (0.23,0.40) | 473731 | 284 (0.06) | 23039 | 83 (0.36) |
| **Acute cardiac events^d^** |  |  |  |  |  |  |  |
| Dysrhythmia | **16.43 (6.03,67.78)** | **<0.001** | 0.21 (0.15,0.28) | 487175 | 80 (0.02) | 23402 | 52 (0.22) |
| Ischemic heart disease | 1.07 (0.38,3.02) | 0.899 | 0.00 (-0.03,0.04) | 479391 | 195 (0.04) | 23229 | 8 (0.03) |
| **All dengue cases versus population-based controls without dengue, aged ≥60 years** | | | | | | | |
| **Composite acute cardiac events** |  |  |  |  |  |  |  |
| Any major-adverse-cardiac-event (MACE)^b^ | **3.62 (2.08,6.73)** | **<0.001** | 0.24 (0.14,0.34) | 654318 | 530 (0.08) | 16584 | 54 (0.33) |
| Any acute cardiac event^c^ | **9.87 (6.53,15.70)** | **<0.001** | 1.25 (1.05,1.44) | 637217 | 869 (0.14) | 16108 | 223 (1.38) |
| **Acute cardiac events^d^** |  |  |  |  |  |  |  |
| Dysrhythmia | **17.29 (9.60,35.21)** | **<0.001** | 0.96 (0.80,1.11) | 673091 | 408 (0.06) | 17108 | 173 (1.01) |
| Ischemic heart disease | **3.78 (2.13,7.17)** | **<0.001** | 0.23 (0.13,0.33) | 658008 | 496 (0.08) | 16679 | 52 (0.31) |

OR> 1 denotes higher odds of a respective composite/individual outcome amongst dengue cases and population-based controls without dengue. As T_0_ was randomly assigned in population-based controls according to the distribution of T_0_ amongst test-positives, changes in the distribution of T_0_ amongst test-positives resulted in differing assignment of T_0_ in population-based controls, and hence differing numbers of population-based controls were filtered out during cohort construction.

Abbreviations: CI, confidence interval; OR, odds ratio; EB, excess burden

^a^ Logistic regression, with overlap weights applied; weights were estimated based on demographic characteristics (age, sex, ethnicity), socioeconomic status (housing type), comorbidities, and healthcare utilisation. EBs were computed by taking the differences in weighted incidences between comparator groups. For subgroup analyses, the number of uninfected controls does not add up to the original number in the main analysis because the filtering steps for the subgroup were performed before assignment of T_0_.

^b^ Major-adverse-cardiac-event (MACE) was defined as the first incidence of myocardial infarction, stroke, ventricular arrhythmia, or sudden cardiac death.

^c^ Any acute cardiac event was taken as a composite of any ischemic heart disease, dysrhythmias, inflammatory heart disease, other cardiac disease, and thrombotic conditions.

^d^ Although cases of new-incident inflammatory heart disease (eg. myocarditis/pericarditis), other cardiac disease (eg. cardiomyopathy/cardiogenic shock) and thrombotic conditions (eg. deep venous thrombosis) were included in the composite of acute cardiac events, separate categories were not computed given the small number of incident cases

^e^ The age 18-39 subgroup was excluded as the relatively lower risk of acute cardiac events in young adults meant that estimation of ORs for several of the cardiac outcomes was not possible, due to zero events in some categories.

**Supplementary Table 3: Odds and excess burden of new-onset acute cardiac events in NS1-positive dengue cases, versus population-based controls without dengue**

| **Outcomes** | **Adjusted odds-ratio, aOR,^a^**  **95% CI** | **p-value (aOR)** | **Excess burden (EB) per-100-persons, 95%CI** | **Controls without dengue (N)** | **Controls without dengue,  with outcome N(%)** | **NS-1 positive dengue cases^b^ (N)** | **NS-1 positive dengue cases^b^  with outcome N(%)** |
| --- | --- | --- | --- | --- | --- | --- | --- |
| **All dengue cases versus population-based controls without dengue** | | | | | | | |
| **Composite acute cardiac events** |  |  |  |  |  |  |  |
| Any major-adverse-cardiac-event (MACE)^c^ | **2.04 (1.15, 3.66)** | **0.016** | 0.04 (0.00, 0.07) | 1560921 | 785 (0.05) | 36989 | 26 (0.07) |
| Any acute cardiac event^d^ | **9.96 (6.98, 14.67)** | **<0.001** | 0.50 (0.41, 0.58) | 1536137 | 1219 (0.079) | 36667 | 198 (0.54) |
| **Acute cardiac events^e^** |  |  |  |  |  |  |  |
| Dysrhythmia | **17.50 (10.53, 31.57)** | **<0.001** | 0.39 (0.32, 0.45) | 1586695 | 536 (0.034) | 37351 | 150 (0.402) |
| Ischemic heart disease | **2.07 (1.14, 3.82)** | **0.017** | 0.03 (0.00, 0.07) | 1565431 | 710 (0.045) | 37035 | 24 (0.065) |

OR> 1 denotes higher odds of a respective composite/individual outcome amongst dengue cases and population-based controls without dengue

Abbreviations: CI, confidence interval; OR, odds ratio; EB, excess burden

^a^ Logistic regression, with overlap weights applied; weights were estimated based on demographic characteristics (age, sex, ethnicity), socioeconomic status (housing type), comorbidities, and healthcare utilisation. EBs were computed by taking the differences in weighted incidences between comparator groups.

^b^ Defined as a DENV-infected case recorded in the national dengue registry with a corresponding positive result for NS1 (non-structural protein 1) antigen recorded within 7 days from the date of notification for DENV-infection.

^C^ Major-adverse-cardiac-event (MACE) was defined as the first incidence of myocardial infarction, stroke, ventricular arrhythmia, or sudden cardiac death.

^d^ Any acute cardiac event was taken as a composite of any ischemic heart disease, dysrhythmias, inflammatory heart disease, other cardiac disease, and thrombotic conditions.

^e^ Although cases of new-incident inflammatory heart disease (eg. myocarditis/pericarditis), other cardiac disease (eg. cardiomyopathy/cardiogenic shock) and thrombotic conditions (eg. deep venous thrombosis) were included in the composite of acute cardiac events, separate categories were not computed given the small number of incident cases

**Supplementary Table 4: Odds and excess burden of new-onset acute myocardial infarction in dengue cases, versus population-based controls without dengue (from** **1^st^ Jan 2017-31^st^ Dec 2021), using a)national healthcare claims data; b)acute myocardial infarction registry data**

| **Outcomes** | **Adjusted odds-ratio, aOR,^a^**  **95% CI** | **p-value (aOR)** | **Excess burden (EB) per-100-persons, 95%CI** | **Controls without dengue (N)** | **Controls without dengue,  with outcome N(%)** | **Dengue cases (N)** | **Dengue cases  with outcome N(%)** |
| --- | --- | --- | --- | --- | --- | --- | --- |
| **All dengue cases versus population-based controls without dengue** | | | | | | | |
| Acute myocardial infarction (defined using registry data)^b^ | **6.50 (2.89, 17.76)** | **<0.001** | 0.09 (0.05, 0.12) | 1284413 | 327 (0.03) | 39088 | 39 (0.10) |
| Acute myocardial infarction (defined using claims data)^c^ | **9.93 (4.47, 27.43)** | **<0.001** | 0.13 (0.09, 0.17) | 1284413 | 323(0.03) | 39088 | 54 (0.14) |

OR> 1 denotes higher odds of a respective composite/individual outcome amongst dengue cases and population-based controls without dengue

Abbreviations: CI, confidence interval; OR, odds ratio; EB, excess burden

^a^ Logistic regression, with overlap weights applied; weights were estimated based on demographic characteristics (age, sex, ethnicity), socioeconomic status (housing type), comorbidities, and healthcare utilisation. EBs were computed by taking the differences in weighted incidences between comparator groups.

^b^ Acute myocardial infarction events were defined as events recorded in the Singapore Myocardial Infarction Registry.

^c^ Acute myocardial infarction events were defined using ICD-10 codes (I21^x^, I22^x^) recorded in national health insurance claims data.

**Supplementary Table 5: Hazards-ratio and excess burden of new-onset acute cardiac events in dengue cases, versus population-based controls without dengue**

| **Outcomes** | **Adjusted hazard-ratio, aHR,^a^**  **95% CI** | **p-value (aHR)** | **Excess burden (EB) per-100-persons, 95%CI** | **Controls without dengue (N)** | **Controls without dengue,  with outcome N(%)** | **Dengue cases (N)** | **Dengue cases  with outcome N(%)** |
| --- | --- | --- | --- | --- | --- | --- | --- |
| **All dengue cases versus population-based controls without dengue** | | | | | | | |
| **Composite acute cardiac events** |  |  |  |  |  |  |  |
| Any major-adverse-cardiac-event (MACE)^b^ | **2.86 (2.21, 3.72)** | **<0.001** | 0.07 (0.04,0.10) | 1560921 | 785 (0.05) | 63642 | 64 (0.10) |
| Any acute cardiac event^c^ | **10.51 (9.28, 11.89)** | **<0.001** | 0.53 (0.47,0.60) | 1536137 | 1219 (0.08) | 62921 | 361 (0.57) |
| **Acute cardiac events^d^** |  |  |  |  |  |  |  |
| Dysrhythmia^e^ | **18.31 (15.64, 21.44)** | **<0.001** | 0.41 (0.35,0.46) | 1586695 | 536 (0.03) | 64306 | 271 (0.42) |
| Ischemic heart disease | **2.99 (2.29, 3.92)** | **<0.001** | 0.06 (0.04,0.09) | 1565431 | 710 (0.05) | 63753 | 60 (0.09) |
| **Ambulatory dengue cases versus population-based controls without dengue^f^** | | | | | | | |
| **Composite acute cardiac events** |  |  |  |  |  |  |  |
| Any major-adverse-cardiac-event (MACE)^b^ | **3.09 (2.28, 4.2)** | **<0.001** | 0.07 (0.04,0.11) | 1560921 | 785 (0.05) | 45307 | 46 (0.10) |
| Any acute cardiac event^c^ | **6.78 (5.67, 8.12)** | **<0.001** | 0.29 (0.23,0.35) | 1536137 | 1219 (0.08) | 44832 | 146 (0.33) |
| **Acute cardiac events^d^** |  |  |  |  |  |  |  |
| Dysrhythmia | **11.76 (9.35, 14.79)** | **<0.001** | 0.21 (0.16,0.26) | 1586695 | 536 (0.03) | 45681 | 100 (0.22) |
| Ischemic heart disease | **3.23 (2.36, 4.41)** | **<0.001** | 0.07 (0.03,0.1) | 1565431 | 710 (0.05) | 45378 | 43 (0.10) |
| **Hospitalised dengue cases versus population-based controls without dengue^f^** | | | | | | | |
| **Composite acute cardiac events** |  |  |  |  |  |  |  |
| Any major-adverse-cardiac-event (MACE)^b^ | **2.00 (1.25, 3.20)** | **0.004** | 0.05 (-0.01,0.11) | 1560921 | 785 (0.05) | 18335 | 18 (0.10) |
| Any acute cardiac event^c^ | **15.01 (12.93, 17.41)** | **<0.001** | 1.11 (0.94,1.27) | 1536137 | 1219 (0.08) | 18089 | 215 (1.19) |
| **Acute cardiac events^d^** |  |  |  |  |  |  |  |
| Dysrhythmia | **27.41 (22.89, 32.81)** | **<0.001** | 0.88 (0.74,1.03) | 1586695 | 536 (0.03) | 18625 | 171 (0.92) |
| Ischemic heart disease | **2.03 (1.26, 3.30)** | **0.004** | 0.05 (-0.01,0.10) | 1565431 | 710 (0.05) | 18375 | 17 (0.09) |
| **Possible secondary dengue cases (IgG positive), versus population-based controls without dengue^g^** | | | | | | | |
| **Composite acute cardiac events** |  |  |  |  |  |  |  |
| Any major-adverse-cardiac-event (MACE)^b^ | **4.34 (2.75, 6.87)** | **<0.001** | 0.10 (0.02-0.18) | 1560921 | 785 (0.05) | 12443 | 19 (0.15) |
| Any acute cardiac event^c^ | **13.77 (11.11, 17.06)** | **<0.001** | 0.68 (0.52-0.85) | 1536137 | 1219 (0.08) | 12220 | 94 (0.77) |
| **Acute cardiac events^d^** |  |  |  |  |  |  |  |
| Dysrhythmia | **22.64 (17.41, 29.43)** | **<0.001** | 0.49 (0.36-0.63) | 1586695 | 536 (0.03) | 12631 | 67 (0.53) |
| Ischemic heart disease | **4.76 (3.01, 7.53)** | **<0.001** | 0.10 (0.02-0.18) | 1565431 | 710 (0.05) | 12484 | 19 (0.15) |
| **Possible primary dengue cases (IgG negative), versus population-based controls without dengue^g^** | | | | | | | |
| **Composite acute cardiac events** |  |  |  |  |  |  |  |
| Any major-adverse-cardiac-event (MACE)^b^ | **2.58 (1.65, 4.03)** | **<0.001** | 0.06 (0.02-0.11) | 1560921 | 785 (0.05) | 22660 | 20 (0.09) |
| Any acute cardiac event^c^ | **10.75 (8.94, 12.94)** | **<0.001** | 0.54 (0.44-0.65) | 1536137 | 1219 (0.08) | 22460 | 130 (0.58) |
| **Acute cardiac events^d^** |  |  |  |  |  |  |  |
| Dysrhythmia | **17.64 (14.03, 22.18)** | **<0.001** | 0.39 (0.30-0.47) | 1586695 | 536 (0.03) | 22818 | 92 (0.40) |
| Ischemic heart disease | **2.55 (1.59, 4.08)** | **<0.001** | 0.06 (0.01-0.10) | 1565431 | 710 (0.05) | 22694 | 18 (0.08) |
| **Dengue cases versus population-based controls without dengue, DENV3-predominant transmission^h^** | | | | | | | |
| **Composite acute cardiac events** |  |  |  |  |  |  |  |
| Any major-adverse-cardiac-event (MACE)^b^ | **3.12 (2.35, 4.14)** | **<0.001** | 0.08 (0.04,0.11) | 1645388 | 818 (0.05) | 49151 | 54 (0.11) |
| Any acute cardiac event^c^ | **10.67 (9.32, 12.22)** | **<0.001** | 0.54 (0.47,0.61) | 1618275 | 1266 (0.08) | 48533 | 284 (0.59) |
| **Acute cardiac events^d^** |  |  |  |  |  |  |  |
| Dysrhythmia | **17.71 (14.91, 21.04)** | **<0.001** | 0.40 (0.34,0.45) | 1673271 | 512 (0.03) | 49693 | 203 (0.41) |
| Ischemic heart disease | **3.23 (2.41, 4.32)** | **<0.001** | 0.07 (0.04,0.10) | 1650133 | 764 (0.05) | 49244 | 50 (0.10) |
| **Dengue cases versus population-based controls without dengue, DENV1/2-predominant transmission^h^** | | | | | | | |
| **Composite acute cardiac events** |  |  |  |  |  |  |  |
| Any major-adverse-cardiac-event (MACE)^b^ | **2.00 (1.07, 3.74)** | **0.030** | 0.03 (-0.02,0.08) | 1248247 | 780 (0.06) | 14491 | 10 (0.07) |
| Any acute cardiac event^c^ | **9.94 (7.87, 12.56)** | **<0.001** | 0.48 (0.36,0.61) | 1231961 | 1119 (0.09) | 14388 | 77 (0.54) |
| **Acute cardiac events^d^** |  |  |  |  |  |  |  |
| Dysrhythmia | **20.35 (15.71, 26.38)** | **<0.001** | 0.45 (0.34,0.57) | 1265720 | 453 (0.04) | 14613 | 68 (0.46) |
| Ischemic heart disease | **2.19 (1.17, 4.10)** | **0.014** | 0.03 (-0.02,0.09) | 1251760 | 709 (0.06) | 14509 | 10 (0.07) |

HR> 1 denotes higher risk of a respective composite/individual outcome amongst dengue cases and population-based controls without dengue

Abbreviations: CI, confidence interval; HR, hazard-ratio; EB, excess burden

^a^ Cox regression, with overlap weights applied; weights were estimated based on demographic characteristics (age, sex, ethnicity), socioeconomic status (housing type), comorbidities, and healthcare utilisation. EBs were computed by taking the differences in weighted incidences between comparator groups.

^b^ Major-adverse-cardiac-event (MACE) was defined as the first incidence of myocardial infarction, stroke, ventricular arrhythmia, or sudden cardiac death.

^c^ Any acute cardiac event was taken as a composite of any ischemic heart disease, dysrhythmias, inflammatory heart disease, other cardiac disease, and thrombotic conditions.

^d^ Although cases of new-incident inflammatory heart disease (eg. myocarditis/pericarditis), other cardiac disease (eg. cardiomyopathy/cardiogenic shock) and thrombotic conditions (eg. deep venous thrombosis) were included in the composite of acute cardiac events, separate categories were not computed given the small number of incident cases

^e^  Of the 271 new-onset dysrhythmia events recorded following acute dengue infection, atrial fibrillation/flutter comprised the majority of events (49.8%, 135/271); other dysrhythmias included sinus bradycardia (N=74), sinus tachycardia (N=54), and other arrhythmias (N=8)

^f^ Hospitalisations attributed to dengue (ICD-10 code A90/A91) occurring within 30 days of a positive dengue test were categorised as dengue hospitalisations; reported dengue cases without a dengue-related hospitalisation were classified as ambulatory cases.

^g^ Acute dengue cases (NS1/IgM positive) with concurrent positive IgG were classified as possible secondary dengue cases; acute dengue cases (NS1/IgM positive) with concurrent negative dengue IgG were classified as possible primary dengue cases. Numbers do not add up to the original number of dengue cases because acute dengue cases (NS1/IgM positive) that were not concurrently tested for dengue IgG were excluded.

^h^ Dengue cases recorded before January 2020 were attributed to DENV1/2; dengue cases recorded after January 2020 onwards were attributed to DENV3. In January 2020, surveillance detected a shift from DENV1/2 to DENV-3, which coincided with a surge in dengue infections; Singapore had not experienced a DENV3 outbreak in the past 30 years. The total number of uninfected population controls differs from the original number in the main cohort (N=1616865) because in the main cohort, T_0_ in uninfected population controls was assigned from a pool of dates ranging from 2017-2023 (matching distribution of the dengue cases); whereas T_0_ for uninfected population controls was assigned from a pool of pre-2020 dates (for comparison against DENV1/2 cases) and for post-2020 dates (for comparison against DENV3 cases), respectively.

**Supplementary Table 6: Odds of acute cardiac events in dengue cases versus population-based controls without dengue, using alternative weighting schemes**

| **Outcomes** | Adjusted odds-ratio, aOR,  95% CI | p-value (aOR) | Excess burden (EB) per-100-persons, 95%CI | Controls without dengue (N) | Controls without dengue,  with outcome N(%) | Dengue cases (N) | Dengue cases  with outcome N(%) |
| --- | --- | --- | --- | --- | --- | --- | --- |
| **Dengue cases versus population-based controls without dengue, inverse-propensity weights^a^** | | | | | | | |
| **Composite acute cardiac events** |  |  |  |  |  |  |  |
| Any major-adverse-cardiac-event (MACE)^b^ | **3.59 (3.32,3.88)** | **<0.001** | 0.13 (0.12,0.14) | 1560921 | 785 (0.05) | 63642 | 64 (0.10) |
| Any acute cardiac event^c^ | **12.00 (11.33,12.72)** | **<0.001** | 0.85 (0.84,0.87) | 1536137 | 1219 (0.08) | 62921 | 361 (0.57) |
| **Acute cardiac events^d^** |  |  |  |  |  |  |  |
| Dysrhythmia | **20.58 (18.91,22.44)** | **<0.001** | 0.65 (0.64,0.66) | 1586695 | 536 (0.03) | 64306 | 271 (0.42) |
| Ischemic heart disease | **3.82 (3.53,4.15)** | **<0.001** | 0.13 (0.12,0.13) | 1565431 | 710 (0.05) | 63753 | 60 (0.09) |
| **Dengue cases versus population-based controls without dengue, doubly robust^e^** | | | | | | | |
| **Composite acute cardiac events** |  |  |  |  |  |  |  |
| Any major-adverse-cardiac-event (MACE)^b^ | **2.82 (2.61,3.06)** | **<0.001** | 0.13 (0.12,0.14) | 1560921 | 785 (0.05) | 63642 | 64 (0.10) |
| Any acute cardiac event^c^ | **9.72 (9.18,10.31)** | **<0.001** | 0.85 (0.84,0.87) | 1536137 | 1219 (0.08) | 62921 | 361 (0.57) |
| **Acute cardiac events^d^** |  |  |  |  |  |  |  |
| Dysrhythmia | **16.66 (15.31,18.17)** | **<0.001** | 0.65 (0.64,0.66) | 1586695 | 536 (0.03) | 64306 | 271 (0.42) |
| Ischemic heart disease | **3.00 (2.76,3.25)** | **<0.001** | 0.13 (0.12,0.13) | 1565431 | 710 (0.05) | 63753 | 60 (0.09) |

^a^ Logistic regression, with inverse propensity weights applied; weights were estimated based on demographic characteristics (age, sex, ethnicity), socioeconomic status (housing type), comorbidities, and healthcare utilisation. EBs were computed by taking the differences in weighted incidences between comparator groups.

^b^ Major-adverse-cardiac-event (MACE) was defined as the first incidence of myocardial infarction, stroke, ventricular arrhythmia, or sudden cardiac death.

^c^ Any acute cardiac event was taken as a composite of any ischemic heart disease, dysrhythmias, inflammatory heart disease, other cardiac disease, and thrombotic conditions.

^d^ Although cases of new-incident inflammatory heart disease (eg. myocarditis/pericarditis), other cardiac disease (eg. cardiomyopathy/cardiogenic shock) and thrombotic conditions (eg. deep venous thrombosis) were included in the composite of acute cardiac events, separate categories were not computed given the small number of incident cases

^e^ Logistic regression, with inverse propensity weights applied; weights were estimated based on demographic characteristics (age, sex, ethnicity), socioeconomic status (housing type), comorbidities, and healthcare utilisation. Explanatory variables used in the propensity score model were added to the outcome variable to prevent model misspecification in either the propensity score model or the outcome model (but not both). EBs were computed by taking the differences in weighted incidences between comparator groups.

**Supplementary Table 7: Baseline characteristics of Singaporean adults with dengue or COVID-19, with standardised-mean-differences before and after overlap weighting**

| **Variables** | **COVID-19 cases (N=1,440,779)** | **Dengue cases (N=62,346)** | **Standardised-mean-difference, baseline** | **COVID-19 cases, weighted** | **Dengue cases, weighted** | **Standardised-mean-difference, post-weighting^‡^** |
| --- | --- | --- | --- | --- | --- | --- |
| **Age, years (S.D)** | 47.82 (17.78) | 48.53 (17.86) | 0.04 | 48.47 (17.88) | 48.47 (17.85) | 0.00 |
| **Age distribution, years** |  |  |  |  |  |  |
| 18-39 years | 563821 (39.13%) | 22702 (36.41%) | 0.06 | 22066 (37.47%) | 21541 (36.58%) | 0.00 |
| 40-59 years | 497043 (34.50%) | 22494 (36.08%) | 0.03 | 20819 (35.36%) | 21214 (36.03%) | 0.00 |
| ≥60 years | 379915 (26.37%) | 17150 (27.51%) | 0.03 | 15998 (27.17%) | 16129 (27.39%) | 0.00 |
| **Gender** |  |  |  |  |  |  |
| Male | 692076 (48.03%) | 33357 (53.50%) | 0.11 | 31362 (53.26%) | 31362 (53.26%) | 0.00 |
| **Ethnicity** |  |  |  |  |  |  |
| Chinese | 1078610 (74.86%) | 49478 (79.36%) | 0.11 | 46588 (79.12%) | 46588 (79.12%) | 0.00 |
| Malay | 114771 (7.97%) | 5115 (8.20%) | 0.01 | 4840 (8.22%) | 4840 (8.22%) | 0.00 |
| Indian | 207064 (14.37%) | 5949 (9.54%) | 0.16 | 5764 (9.79%) | 5764 (9.79%) | 0.00 |
| Others^§^ | 40334 (2.80%) | 1804 (2.89%) | 0.01 | 1692 (2.87%) | 1692 (2.87%) | 0.00 |
| **Housing type** |  |  |  |  |  |  |
| 1-2 room public housing | 64589 (4.48%) | 2409 (3.86%) | 0.03 | 2314 (3.93%) | 2314 (3.93%) | 0.00 |
| 3-room public housing | 219076 (15.21%) | 8116 (13.02%) | 0.06 | 7804 (13.25%) | 7804 (13.25%) | 0.00 |
| 4-room public housing | 505760 (35.10%) | 15974 (25.62%) | 0.22 | 15456 (26.25%) | 15456 (26.25%) | 0.00 |
| 5-room public housing | 570942 (39.63%) | 24183 (38.79%) | 0.02 | 23151 (39.32%) | 23151 (39.32%) | 0.00 |
| Private housing/Others | 80412 (5.58%) | 11664 (18.71%) | 0.35 | 10159 (17.25%) | 10159 (17.25%) | 0.00 |
| **Comorbidity burden (Charlson Comorbidity Index, CCMI)^↑^** |  |  |  |  |  |  |
| No comorbidities (CCMI=0) | 1183792 (82.16%) | 52513 (84.23%) | 0.06 | 49519 (84.1%) | 49519 (84.1%) | 0.00 |
| Mild comorbidity burden (CCMI 1-3) | 213982 (14.85%) | 8090 (12.98%) | 0.06 | 7745 (13.15%) | 7701 (13.08%) | 0.00 |
| Moderate-severe comorbidity burden (CCMI >3) | 43005 (2.98%) | 1743 (2.80%) | 0.01 | 1619 (2.75%) | 1663 (2.82%) | 0.00 |
| **Comorbidities** |  |  |  |  |  |  |
| Diabetes | 12502 (0.87%) | 333 (0.53%) | 0.05 | 322 (0.55%) | 322 (0.55%) | 0.00 |
| Dyslipidemia | 251396 (17.45%) | 7761 (12.45%) | 0.15 | 7473 (12.69%) | 7473 (12.69%) | 0.00 |
| Ischemic heart disease | 3060 (0.21%) | 162 (0.26%) | 0.01 | 152 (0.26%) | 152 (0.26%) | 0.00 |
| Cerebrovascular disease | 33339 (2.31%) | 1317 (2.11%) | 0.01 | 1255 (2.13%) | 1255 (2.13%) | 0.00 |
| **Healthcare utilisation** |  |  |  |  |  |  |
| Prior hospitalisation/emergency department visit in the past 1 year | 720729 (50.02%) | 34808 (55.83%) | 0.12 | 32697 (55.53%) | 32697 (55.53%) | 0.00 |

Data are n or n (%). Number of dengue-infected cases do not add up to N=65,207 because individuals with SARS-CoV-2 infection within 90d of dengue infection were additionally excluded.

^§^Includes individuals of other ethnicities or mixed ethnicities.

^↑^Comorbidity burden was defined using the Charlson Comorbidity Index (CCMI), which consists of the following comorbidities: myocardial infarction, chronic heart failure, peripheral vascular disease, cerebrovascular accident, dementia, chronic obstructive pulmonary disease, connective tissue disease, peptic ulcer disease, diabetes mellitus, hemiplegia, liver disease, moderate to severe renal impairment, solid tumor, leukemia, human immunodeficiency virus (HIV) infection with AIDS.

^‡^ Standardised-mean-difference, SMD after overlap weighting of dengue cases and COVID-19 cases, weighted from original samples

**Supplementary Table 8: Odds of new-onset acute cardiac events in dengue cases versus COVID-19 cases**

| **Outcomes** | Adjusted odds-ratio, aOR,^a^  95% CI | p-value (aOR) | Excess burden (EB) per-100-persons, 95%CI | COVID-19 cases (N) | COVID-19 cases,  with outcome N(%) | Dengue cases (N) | Dengue cases  with outcome N(%) |
| --- | --- | --- | --- | --- | --- | --- | --- |
| **Composite acute cardiac events** |  |  |  |  |  |  |  |
| Any major-adverse-cardiac-event (MACE)^b^ | **2.29 (1.46,3.70)** | **<0.001** | 0.06 (0.03,0.09) | 1407047 | 609 (0.04) | 60858 | 63 (0.10) |
| Any acute cardiac event^c^ | **4.70 (3.65,6.14)** | **<0.001** | 0.45 (0.38,0.52) | 1390205 | 1584 (0.11) | 60174 | 345 (0.57) |
| **Acute cardiac events^d^** |  |  |  |  |  |  |  |
| Dysrhythmia | **5.47 (4.02,7.60)** | **<0.001** | 0.35 (0.29,0.41) | 1420831 | 1015 (0.07) | 61488 | 262 (0.43) |
| Ischemic heart disease | **2.27 (1.43,3.71)** | **<0.001** | 0.05 (0.02,0.08) | 1409419 | 578 (0.04) | 60961 | 59 (0.10) |

OR> 1 denotes higher odds of a respective composite/individual outcome amongst dengue cases and COVID-19 cases

Abbreviations: CI, confidence interval; OR, odds ratio; EB, excess burden

^a^ Logistic regression, with overlap weights applied; weights were estimated based on demographic characteristics (age, sex, ethnicity), socioeconomic status (housing type), comorbidities, and healthcare utilisation. EBs were computed by taking the differences in weighted incidences between comparator groups.

^b^ Major-adverse-cardiac-event (MACE) was defined as the first incidence of myocardial infarction, stroke, ventricular arrhythmia, or sudden cardiac death.

^c^ Any acute cardiac event was taken as a composite of any ischemic heart disease, dysrhythmias, inflammatory heart disease, other cardiac disease, and thrombotic conditions.

^d^ Although cases of new-incident inflammatory heart disease (eg. myocarditis/pericarditis), other cardiac disease (eg. cardiomyopathy/cardiogenic shock) and thrombotic conditions (eg. deep venous thrombosis) were included in the composite of acute cardiac events, separate categories were not computed given the small number of incident cases

**Supplementary Table 9: Odds of negative outcome control (bronchitis) in dengue cases versus population-based controls without dengue**

| **Outcomes** | Adjusted odds-ratio, aOR,^a^  95% CI | p-value (aOR) | Excess burden (EB) per-100-persons, 95%CI | Controls without dengue (N) | Controls without dengue,  with outcome N(%) | Dengue cases (N) | Dengue cases  with outcome N(%) |
| --- | --- | --- | --- | --- | --- | --- | --- |
| **Dengue cases versus population-based controls without dengue** | | | | | | | |
| **Negative outcome control** |  |  |  |  |  |  |  |
| Acute bronchitis | 0.64 (0.03,9.11) | 0.733 | 0.00 (-0.01,0.00) | 1614891 | 54 (0.00) | 65149 | 1 (0.00) |

OR> 1 denotes higher odds of a respective composite/individual outcome amongst dengue cases and population-based controls without dengue

Abbreviations: CI, confidence interval; OR, odds ratio; EB, excess burden

^a^ Logistic regression, with overlap weights applied; weights were estimated based on demographic characteristics (age, sex, ethnicity), socioeconomic status (housing type), comorbidities, and healthcare utilisation. EBs were computed by taking the differences in weighted incidences between comparator groups.

**Supplementary Figure 1: Cohort construction flowchart (dengue versus COVID-19)**

**Dengue cases versus COVID-19 cases**

**
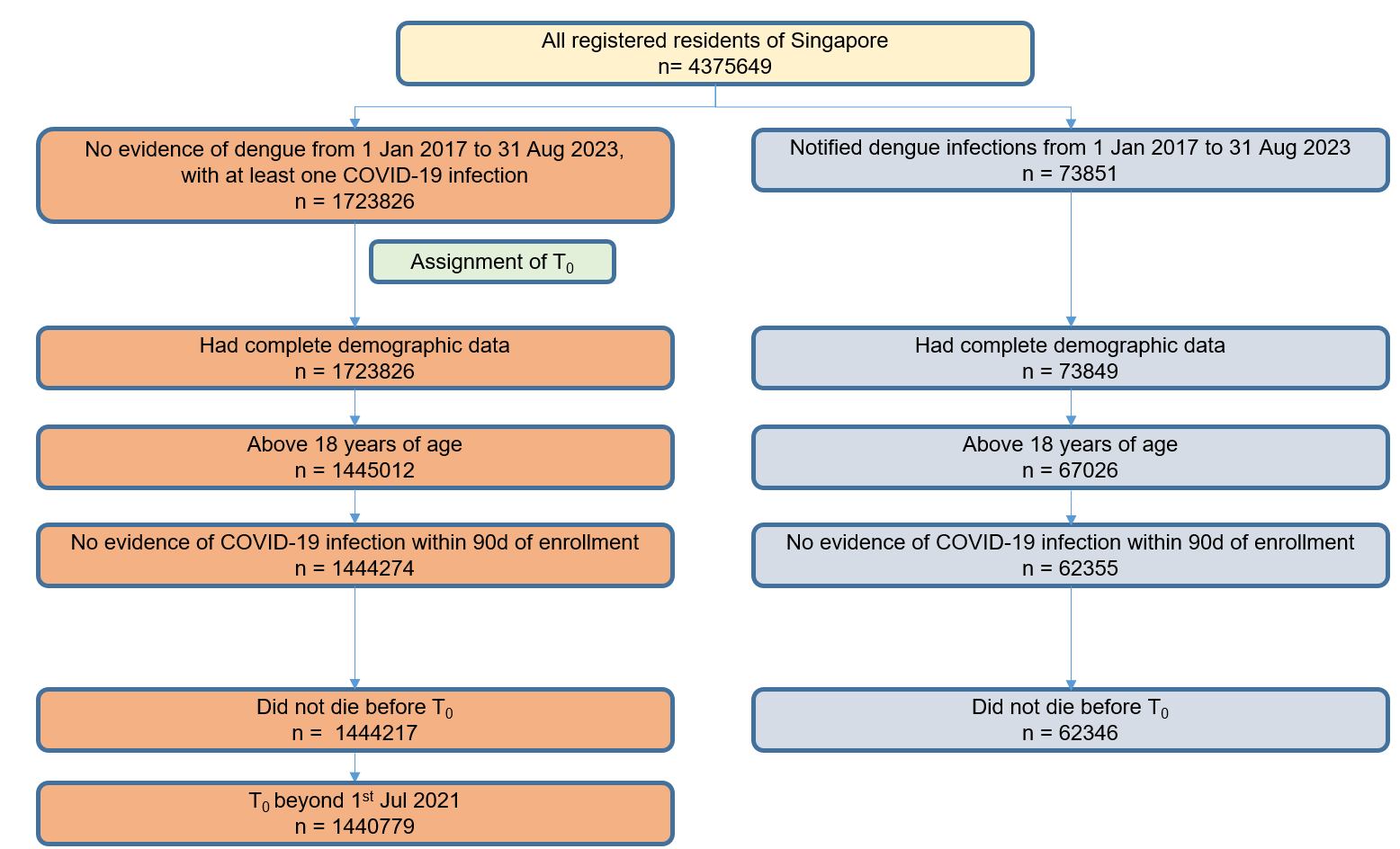
**

# **Supplementary Material: List of ICD-10 codes used for cardiovascular diagnoses of interest**

^x^ denotes that all other subcodes of the listed parent ICD-10 code were included

| **Pre-specified cardiovascular outcomes**  **(subcategories:**  **Dysrhythmia; ischemic heart disease)** | **Outcome** | **ICD 10 Code** | **Description** |
| --- | --- | --- | --- |
|  | **Dysrhythmia** |  |  |
|  | Atrial fibrillation | I48^x^ | Atrial fibrillation and flutter |
|  | Sinus tachycardia | I47^x^ | Paroxysmal tachycardia |
|  |  | R00.0 | Tachycardia, unspecified |
|  | Sinus bradycardia | R00.1 | Bradycardia, unspecified |
|  | Other arrhythmias | I44^x^ | Atrioventricular and left bundle-branch block |
|  |  | R00.2 | Palpitations |
|  |  | R00.8 | Other abnormalities of heart beat |
|  |  | R00.9 | Unspecified abnormalities of heart beat |
|  |  | I45^x^ | Other conduction disorders |
|  | **Inflammatory heart disease** |  | Due to low incidence of new-incident events in this category, this category was counted in the composite of acute cardiovascular events but not evaluated individually. |
|  |  | I49^x^ | Other cardiac arrhythmias |
|  | Pericarditis | I30^x^ | Acute pericarditis |
|  |  | B33.23 | Viral pericarditis |
|  | Myocarditis | I51.4 | Myocarditis, unspecified |
|  |  | B33.20 | Viral carditis, unspecified |
|  |  | B33.21 | Viral endocarditis |
|  |  | B33.22 | Viral myocarditis |
|  |  | B33.24 | Viral cardiomyopathy |
|  |  | I40^x^ | Acute myocarditis |
|  | **Ischemic heart disease** |  |  |
|  | Myocardial infarction | I21^x^ | Acute myocardial infarction |
|  |  | I22^x^ | Subsequent ST elevation (STEMI) and non-ST elevation (NSTEMI) myocardial infarction |
|  | Acute coronary disease | I24^x^ | Other acute ischemic heart diseases |
|  |  | I25.10 | Atherosclerotic heart disease of native coronary artery without angina pectoris |
|  |  | I25.11^x^ | Atherosclerotic heart disease of native coronary artery with angina pectoris |
|  | Ischemic cardiomyopathy | I25.5 | Ischemic cardiomyopathy |
|  | Angina | I20^x^ | Angina pectoris |
|  | **Other heart conditions** |  | Due to low incidence of new-incident events in this category, this category was counted in the composite of acute cardiovascular events but not evaluated individually. |
|  | Cardiomyopathy | I42^x^ | Cardiomyopathy |
|  | Cardiac arrest | I46^x^ | Cardiac arrest |
|  | Cardiogenic shock | R57.0 | Cardiogenic shock |
|  | **Thrombotic conditions** |  | Due to low incidence of new-incident events in this category, this category was counted in the composite of acute cardiovascular events but not evaluated individually. |
|  | Pulmonary embolism | I26^x^ | Pulmonary embolism |
|  | Deep venous thrombosis | I80.1^x^ | Phlebitis and thrombophlebitis of femoral vein |
|  |  | I80.2^x^ | Phlebitis and thrombophlebitis of other and unspecified deep vessels of lower extremities |
|  |  | I81 | Portal vein thrombosis |
|  |  | I82 ^x^ | Other venous embolism and thrombosis |
|  |  | I67.6 | Nonpyogenic thrombosis of intracranial venous system |
|  | Superficial venous thrombosis | I80.0^x^ | Phlebitis and thrombophlebitis of superficial vessels of lower extremities |
|  |  | I80.3 | Phlebitis and thrombophlebitis of lower extremities, unspecified |
|  |  | I80.8 | Phlebitis and thrombophlebitis of other sites |
|  |  | I80.9 | Phlebitis and thrombophlebitis of unspecified site |
|  | Arterial thromboses | I74^x^ | Arterial embolism and thrombosis |
